# miR-221/222 induce instability of p53 By downregulating deubiquitinase YOD1 in acute myeloid leukemia

**DOI:** 10.1038/s41420-023-01537-4

**Published:** 2023-07-15

**Authors:** Han Zhong Pei, Zhiyong Peng, Xiaomei Zhuang, Xiaobo Wang, Bo Lu, Yao Guo, Yuming Zhao, Dengyang Zhang, Yunjun Xiao, Tianshun Gao, Liuting Yu, Chunxiao He, Shunjie Wu, Suk-Hwan Baek, Zhizhuang Joe Zhao, Xiaojun Xu, Yun Chen

**Affiliations:** 1 Department of Hematology, The Seventh Affiliated Hospital, Sun Yat-sen University, Shenzhen, 518107 Guangdong China; 2 Nanfang-Chunfu Children’s Institute of Hematology, Taixin Hospital, Dongguan, Guangdong China; 3 Department of Biochemistry & Molecular Biology, College of Medicine, Yeungnam University, 170 Hyeonchung-ro, Nam-gu, Daegu 42415 South Korea; 4 Department of Pathology, University of Oklahoma Health Sciences Center, 940 Stanton L. Young Blvd., BMSB 451, Oklahoma City, OK 73104 USA

**Keywords:** Acute myeloid leukaemia, Checkpoint signalling

## Abstract

Acute myeloid leukemia (AML) is a hematological malignancy characterized by the impaired differentiation and uncontrolled proliferation of myeloid blasts. Tumor suppressor p53 is often downregulated in AML cells via ubiquitination-mediated degradation. While the role of E3 ligase MDM2 in p53 ubiquitination is well-accepted, little is known about the involvement of deubiquitinases (DUBs). Herein, we found that the expression of YOD1, among several DUBs, is substantially reduced in blood cells from AML patients. We identified that YOD1 deubiqutinated and stabilized p53 through interaction via N-terminus of p53 and OTU domain of YOD1. In addition, expression levels of YOD1 were suppressed by elevated miR-221/222 in AML cells through binding to the 3′ untranslated region of YOD1, as verified by reporter gene assays. Treatment of cells with miR-221/222 mimics and inhibitors yielded the expected effects on YOD1 expressions, in agreement with the negative correlation observed between the expression levels of miR-221/222 and YOD1 in AML cells. Finally, overexpression of YOD1 stabilized p53, upregulated pro-apoptotic p53 downstream genes, and increased the sensitivity of AML cells to FLT3 inhibitors remarkably. Collectively, our study identified a pathway connecting miR-221/222, YOD1, and p53 in AML. Targeting miR-221/222 and stimulating YOD1 activity may improve the therapeutic effects of FLT3 inhibitors in patients with AML.

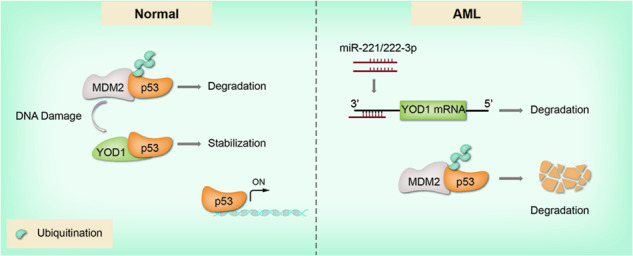

## Introduction

Acute myeloid leukemia (AML) is a genetically heterogeneous and clonal hematopoietic stem cell malignancy characterized by chromosomal abnormalities, recurrent gene mutations, and/or epigenetic modifications affecting chromatin structures [1, 2]. Although many advances have been made in the development of therapeutic drugs to treat the disease [3–7], the clinical outcomes are still undesirable, especially for elderly patients [8–10].

As the most important tumor suppressor, p53 plays a key role in protecting genome integrity and preventing the occurrence of tumors [11]. Unlike other types of cancers, AML often bears wild-type p53, which is genetically intact but suppressed through various mechanisms [2, 12]. Suppression of p53 promotes cell proliferation, leading to the accumulation of DNA damage and subsequent malignant transformation of hematopoietic stem cells [13]. E3 ligase MDM2 plays a key role in controlling the protein levels of p53 via the ubiquitination pathway. MDM2 is usually inhibited by the tumor suppressor ARF, but this inhibition is cancelled in most cases of AML owing to inactivation of ARF [14]. In addition, hyperactivation of the PI3K/AKT and RAS/RAF/MEK/ERK pathways also enhances the function of MDM2 by regulating its subcellular localization and translation in AML cells [15, 16]. Furthermore, as an important transcription factor that regulates survival signals in AML cells, STAT5 can cause instability of p53 by activating MDM2 [17]. Therefore, the downregulation of p53 by ubiquitination-mediated proteasomal degradation is an important regulatory mechanism underlying AML development [18]. Ubiquitination is a reversible process controlled by the coordinated action of ubiquitinating and deubiquitinating enzymes [19]. Studies on p53 ubiquitination in AML mainly revolve around changes in the activity of E3 ligase MDM2, and little is known about the involvement of deubiquitinases (DUBs).

The human genome encodes nearly 100 DUBs divided into seven different families: ubiquitin-specific proteases (USPs), ovarian tumor (OTU) proteases, ubiquitin C-terminal hydrolases (UCHs), Machado–Joseph Disease (MJD) DUBs, motif interacting with ubiquitin (MIU)-containing novel DUB family (MINDY), recently discovered zinc finger-containing ubiquitin peptidase 1 (ZUP1), and JAB1/MPN/MOV34 metalloprotease DUBs (JAMMs) [20]. DUBs regulate the stability, localization, and activity of proteins involved in various signaling pathways, and their role in cancer development varies according to substrate specificities [21]. Since the initial discovery of the role of USP7 in stabilizing the p53 protein [22], several other DUBs, including OTUD1, OTUD5, USP3, USP10, and OTUB1, have been shown to deubiquitinate and stabilize p53 [23–27]. In this study, we analyzed the expression of several candidate DUBs and found that YOD1 was markedly suppressed in AML patient samples compared with that in normal samples. We then confirmed that YOD1 interacts with p53 and stabilizes p53 via deubiquitination. We further demonstrated that the reduced expression of YOD1 was caused by overexpression of miR-221/222 and revealed that YOD1 overexpression augments the inhibitory effects of FLT3 tyrosine kinase inhibitors (TKIs) in AML cells.

## Results

### YOD1 stabilizes p53 by deubiquitination

**Fig. 1:**
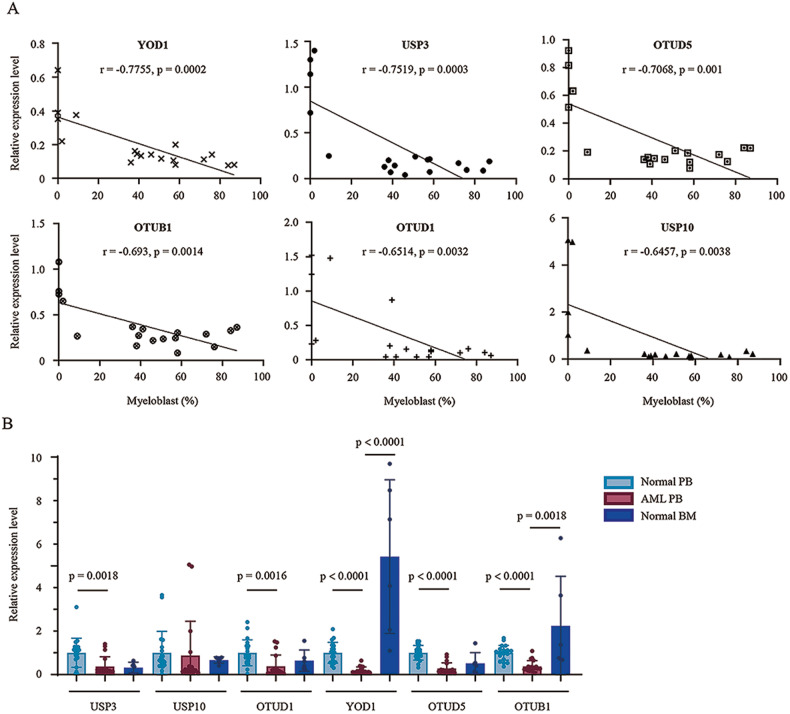
Relative expression levels of representative DUBs in normal and AML blood. **A** Peripheral blood leukocytes were isolated by centrifugation after red blood cell lysis from AML patients (*n* = 18). Relative expression levels of DUBs in reference to GAPDH were determined by qPCR using the 2ΔΔCT method and correlated with the peripheral blood myeloblast percentages. **B** qPCR analysis of selected DUB expressions in leukocytes from healthy blood (*n* = 20), AML blood (*n* = 18) and healthy bone marrow (*n* = 6). Relative expression levels of six DUBs in reference to GAPDH were determined by qPCR using the 2ΔΔCT method. Error bars denote standard deviation. Pearson’s correlation coefficients and *p* values are indicated.

Initially, we isolated leukocytes from 18 AML patients. To investigate the involvement of DUBs in AML, we analyzed the expression of 16 such enzymes in peripheral blood cells using qPCR. These DUBs have been shown to stabilize p53 [23**–**35]. The correlation between DUB expression and the myeloblast percentage is shown in Fig. 1A and Supplementary Fig. 1. Six of these DUBs were negatively correlated with myeloblasts, including USP3, USP10, OTUD1, YOD1, OTUD5, and OTUB1 (Fig. 1A). To confirm whether these genes are specifically under-expressed in AML patients, we collected leukocytes from healthy peripheral blood (*n* = 20) and bone marrow (*n* = 6) and compared them with those from the peripheral blood of AML patients. qPCR data revealed that YOD1 and OTUB1 decreased in AML samples, with YOD1 showing the most significant changes (Fig. 1B). We believe that the decreased YOD1 expression in AML leukocytes was caused by the diminished expression of YOD1 in leukemic myeloblasts. It should be noted that no difference was observed in p53 expression at the mRNA level (Supplementary Fig. 2A), but the protein level of p53 was significantly reduced in AML patient samples (Supplementary Fig. 3).

**Fig. 2:**
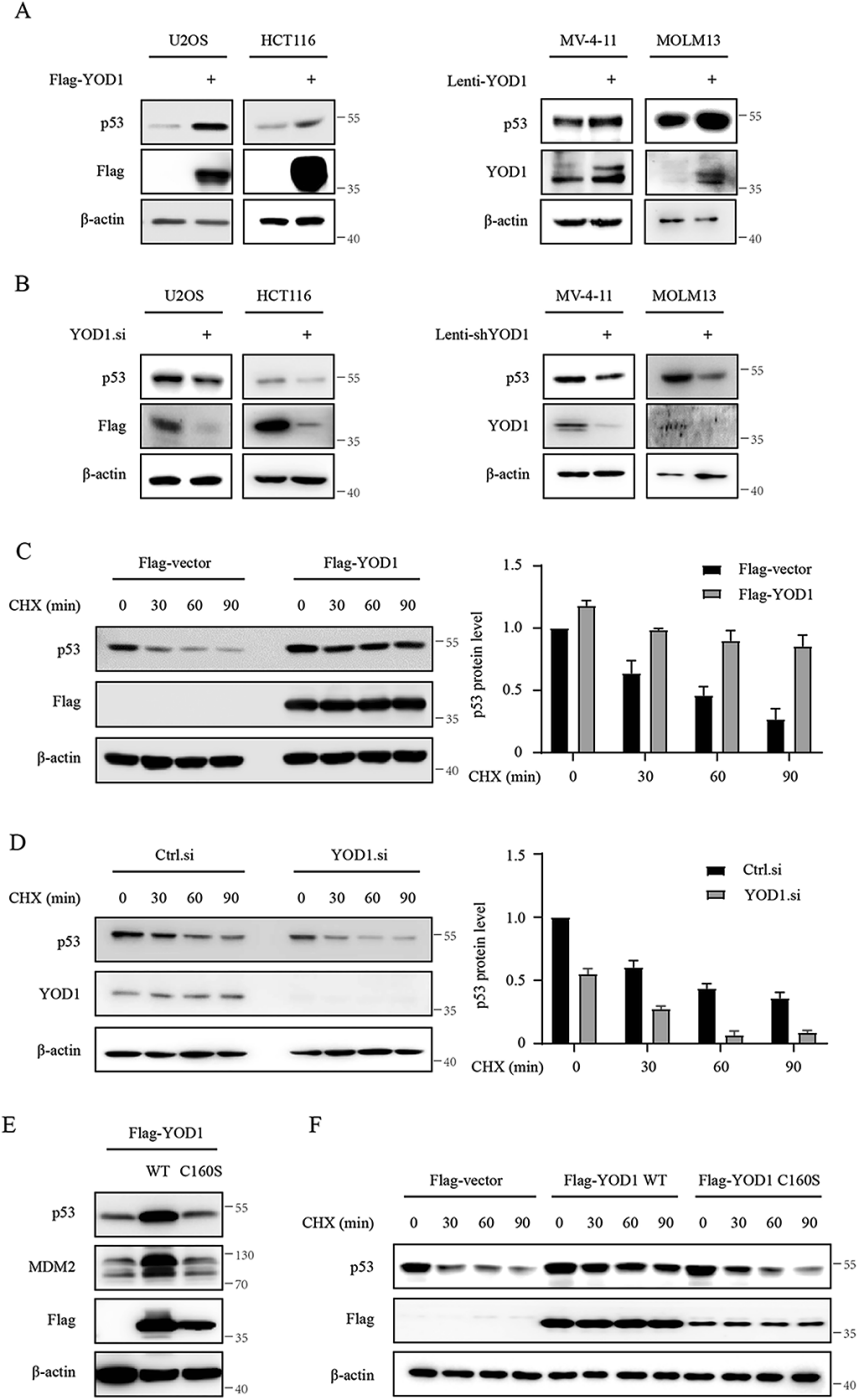
Altered expression of YOD1 affects p53 protein levels. **A** U2OS and HCT116 cells were transfected with Flag-vector or Flag-YOD1 for 36 h, while AML cell lines MV-4-11 and MOLM13 cells were infected with control or YOD1 lentivirus for 96 h. Cell lysates were subjected to immunoblotting with specified antibodies. **B** U2OS and HCT116 cells were transfected with control or YOD1 siRNA for 36 h, and MV-4-11 and MOLM13 cells were infected with control or YOD1 shRNA lentivirus for 96 h. This was followed by immunoblotting assays. **C**, **D** U2OS cells were transfected with Flag-vector or Flag-YOD1 for 36 h and then treated with 50 μg/ml cycloheximide for the indicated periods of time before extraction. Relative protein levels of p53 were determined by densitometric analyses with β-actin as a reference. **E**, **F** Catalytic inactivation of YOD1 by the C160S point mutation diminishes its effects on p53 expression. U2OS cells were transfected with the catalytically inactive C160S mutant form of YOD1 and treated as above. Representative results are shown from triplicate experiments with similar results.

To define the direct role of YOD1 in the regulation of p53, we altered the expression of YOD1 in U2OS, HCT116, MV-4–11, and MOLM13 cells. These cells all bear wild-type p53, and the latter two are AML cell lines. As shown in Fig. 2, overexpression of wild-type YOD1 significantly increased the protein level of p53 (Fig. 2A). In contrast, knockdown of YOD1 using siRNA or lentiviral shRNA displayed opposite effects (Fig. 2B). The effects of altered YOD1 expression on p53 protein were more pronounced when cells were treated with CHX, which inhibits the synthesis of new proteins via translation (Fig. 2C and D). Note that p53 was not affected at the mRNA level by the manipulation of YOD1 expression (Supplementary Fig. 2B and C). We further expressed a catalytically inactive mutant form of YOD1 for overexpression in U2OS cells. The DUB OUT subfamily catalyzes deubiquitination via a conserved cysteine residue [36]. YOD1 corresponded to cysteine 160 (C160). Thus, we generated C160S mutant YOD1 and overexpressed it in U2OS cells. As expected, YOD1-C160S did not affect p53 expression (Fig. 2E and F). These results indicate that YOD1 stabilizes the p53 protein in a catalytic activity-dependent manner.

**Fig. 3:**
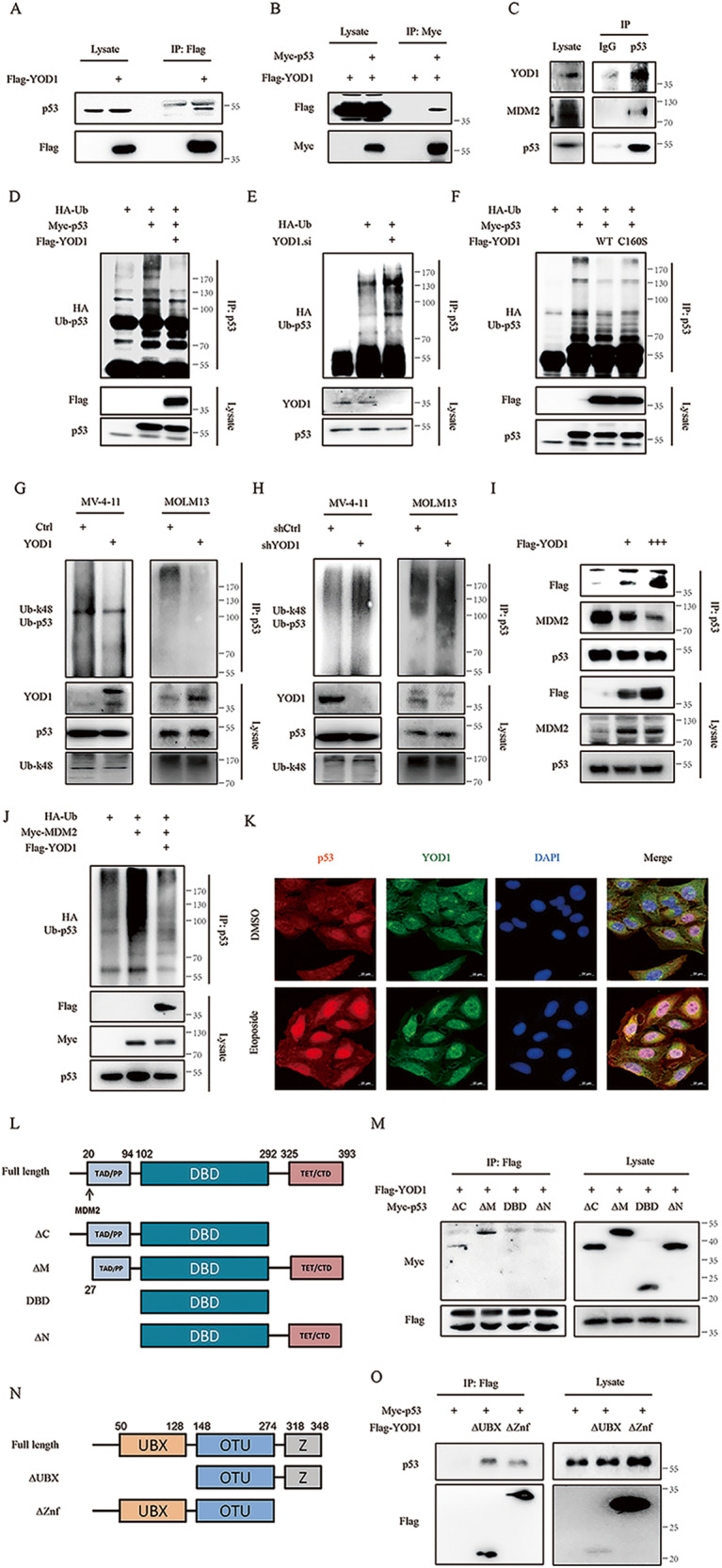
Altered expression of YOD1 affects ubiquitination of p53. **A**, **B** YOD1 interacts with p53. U2OS cells were transfected with indicated cDNA constructs for 36 h. Cell lysates were subjected to immunoprecipitation and then immunoblotting with specified antibodies. **C** p53 forms a complex with YOD1 and MDM2. MV-4-11 cell lysates were immunoprecipitated with anti-p53 antibody, and the immunoprecipitates were subjected to immunoblotting with specified antibodies. **D**, **E** Overexpression of YOD1 reduces ubiquitination of p53 while knockdown of YOD1 enhances the ubiquitination. Note that transfected U2OS cells were treated with 5 μM MG132 for 4 h before extraction. **F**, **G** AML cell lines MV-4-11 and MOLM13 were infected with lentiviral YOD1 (**F**) or YOD1 shRNA (**G**) for 96 h and treated with MG132 for 4 h. Cell lysates were subjected to ubiquitination assay and then IB with specified antibodies. **H** U2OS cells were co-transfected with the indicated cDNA constructs for 36 h, followed by UB and IB assay. **I** 293 T cells were transfected with Flag-YOD1 plasmid for 36 h. Cell lysates were subjected to immunoprecipitation with p53 antibody, and then immunoblotting with specified antibodies. **J** U2OS cell were co-transfected with the indicated cDNA constructs for 36 h, and cell lysates were subjected to ubiquitination assays. **K** p53 and YOD1 immunofluorescence staining of U2OS. Cells were treated with DMSO or Etoposide for 4 h. Bar 20 μm. **L** Schematic diagram of Myc-tagged full length and domain-truncated mutant forms of p53. (M) 293 T cells were transfected with Myc-p53 domain-truncated mutants and Flag-YOD1 for 36 h. Cell lysates were subjected to immunoprecipitation and then immunoblotting with specified antibodies. **N** Schematic diagram of Flag-tagged full length and domain-truncated mutant forms of YOD1. **O** Cells were transfected with Flag-YOD1 domain-truncated mutants and Myc-p53 for 36 h. Cell lysates were subjected to immunoprecipitation with anti-Flag targeted-beads and immunoblotting with specified antibodies. Representative results are shown from triplicate experiments with similar results.

We further used IP to investigate the potential interaction between YOD1 and p53. For this purpose, U2OS cells were transfected with the control empty vector or plasmid carrying Flag-YOD1, and cell extracts were immunoprecipitated using an anti-FLAG tag antibody. Immunoblotting analysis with an anti-p53 antibody revealed the presence of p53 in the immunoprecipitates obtained from cells transfected with Flag-YOD1, but not the empty vector (Fig. 3A). We further co-transfected U2OS cells with Flag-YOD1 and Myc-p53 and performed immunoprecipitation using an anti-Myc tag antibody. As expected, we detected clear binding of Myc-p53 to Flag-YOD1 (Fig. 3B). In addition, we detected an interaction and colocalization of endogenous YOD1 and p53 in MV-4–11 cells (Fig. 3C and Supplementary Fig. 4). Taken together, these data indicated a specific interaction between YOD1 and p53. Presumably, this may lead to the deubiquitination of p53 by YOD1. To determine if this was the case, U2OS cells were co-transfected with plasmids carrying HA-Ub, Myc-p53, and Flag-YOD1 and then treated with the proteasome inhibitor MG132. Immunoblotting analysis of anti-p53 immunoprecipitates revealed a significant reduction in p53 ubiquitination (Fig. 3D). Similar results were observed in HCT116 cells, in which ubiquitination of endogenous p53 was analyzed (Supplementary Fig. 5A). We further analyzed p53 ubiquitination in cells transfected with YOD1 siRNA or a catalytically inactive C160S YOD1 mutant. As expected, the knockdown of YOD1 increased p53 ubiquitination, while overexpression of the mutant showed no effect (Fig. 3E, F and Supplementary Fig. 5B), indicating that the protein level of catalytically active YOD1 is responsible for the changes in p53 ubiquitination. Ubiquitin can be continuously linked to one of the seven lysine residues or to the N-terminal methionine of the previous ubiquitin molecule to form a poly-ubiquitin (poly-Ub) chain. The formation of the K48 poly-Ub chain is closely related to ubiquitination and degradation [19]. Therefore, we used HA-Ub (K48) and HA-Ub (K63), which retain only a single ubiquitination site, to test whether YOD1 displays site-specificity. In U2OS cells, K48-linked but not K63-linked ubiquitination of p53 was reduced upon YOD1 overexpression and increased after YOD1 knockdown (Supplementary Fig. 6A, B). With AML cell lines MV-4–11 and MOLM13, the ubiquitination analysis also demonstrated that p53 k48-poly ubiquitination was significantly reduced in Lenti-YOD1-infected cells but enhanced in Lenti-shYOD1-infected cells (Fig. 3G, H). It is conceivable that YOD1 preferentially deubiquitinates the K48 poly-Ub chain of p53, thereby preventing its degradation.

E3 ligase MDM2 reduces the protein level of p53 by inducing ubiquitination. Interestingly, we found that YOD1 transfection inhibited the p53-MDM2 interaction (Fig. 3I) and consequently MDM2-induced p53 ubiquitination (Fig. 3J). It is known that the p53 stability is enhanced during DNA damage. To examine the role of YOD1 in the process, we induced DNA damage by treating cells with etoposide and detected colocalization of p53 and YOD1 in the nucleus (Fig. 3K), suggesting a protective role of YOD1 in stabilizing p53 during DNA damage. To identify domains in p53 that interact with YOD1, we constructed four p53 mutants, including C-segment deletion (△C), MDM2 binding site deletion (△M), DBD domain alone (DBD), and N-terminal deletion (△N) (Fig. 3L). Through immunoprecipitation, we found that Flag-YOD1 could bind △C and △M but not DBD or △N mutants (Fig. 3M). We also constructed two YOD1 mutants with the deletion of the C-terminal UBX domain (△UBX) and the N-terminal Znf domain (△Znf) (Fig. 3N). Immunoprecipitation assays showed that both mutants retained the ability to interact with p53 (Fig. 3O). These results demonstrate that YOD1 interacts with p53 through the N-terminus of p53 and the OTU domain of YOD1.

### YOD1 is a target of miR-221/222

**Fig. 4:**
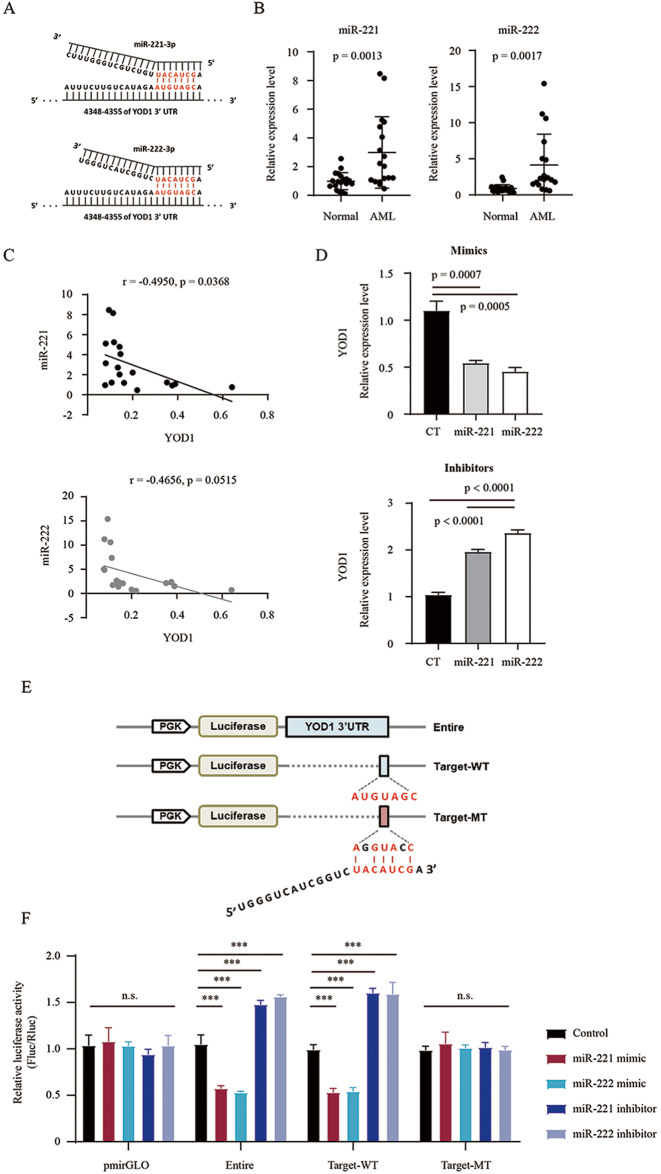
Negative correlation between YOD1 and miR-221/222 expressions and identification of a miR-221/222 target site at 3′ UTR of YOD1. **A** Potential base pairing of YOD1 3′ UTR with miR-221/222-3p (red). **B** Expression of miR-221/222 in leukocytes from AML patients and healthy donors was detected using stem-loop RT-qPCR. (C) Negative correlation between YOD1 and miR-221/222 expression in peripheral blood leukocytes from patients with AML and healthy donors. Pearson’s correlation coefficients and *p* values are indicated. **D** U2OS cells were transfected with miRNA inhibitors or mimics, and YOD1 expression was analyzed via qPCR. **E** Schematic diagram of pmirGLO luciferase constructs containing the entire 3′ UTR of YOD1 (Entire), partial wild-type fragment (Target-WT), and partial mutant fragment (Target-MT). The sequence of miR-222-3p is shown. **F** Luciferase reporter constructs and miRNA mimics or inhibitor were used to transfect U2OS cells. Firefly luciferase (Fluc) and renal luciferase (Rluc) in the cell extracts were used to calculate the relative luciferase activity. *p* < 0.001 (**), *p* < 0.0001 (***) and *p* > 0.05 (ns). Representative results are shown from triplicate experiments with similar results.

It has been reported that miR-221/222 is overexpressed in AML and confers unfavorable prognosis [37]. By analyzing the TargetScanHuman 7.2 database, we found a binding site for miR-221/222 in the 3′ UTR of YOD1 (Fig. 4A). Furthermore, qPCR analysis demonstrated a significantly increased level of miR-221/222 expression in leukocytes from AML patients compared to that in healthy samples (Fig. 4B). Interestingly, we also found a negative correlation between the expression of miR-221/222 and YOD1 (r = −0.4550 and −0.4656, Fig.4C). To confirm the suppressive effects of miR-221/222 on YOD1 expression, we treated U2OS cells with miR-221/222 mimics and inhibitors. While the mimics inhibited YOD1 expression, the inhibitors displayed the opposite effects (Fig. 4D). To verify that miR-221/222 directly targeted YOD1, we performed luciferase reporter assays. We inserted the entire YOD1 3′ UTR or a fragment containing the miR-221/222 target site with the wild-type (Target-WT) or a mutant sequence (Target-MT) into the pmirGLO dual-luciferase plasmid (Fig. 4E). Luciferase activity was analyzed upon co-transfection of the reporter constructs with miR-221/222 mimics or inhibitors. As shown in Fig. 4F, relative luciferase activity from the 3′ UTR and Target-WT constructs was inhibited by miR-221/222 mimics but enhanced by their inhibitors, while that from the control vector and Target-MT constructs was not affected. These data indicate that miR-221/222 targets YOD1 at the predicted site.

### MiR-221/222 enhances p53 ubiquitination by targeting YOD1

**Fig. 5:**
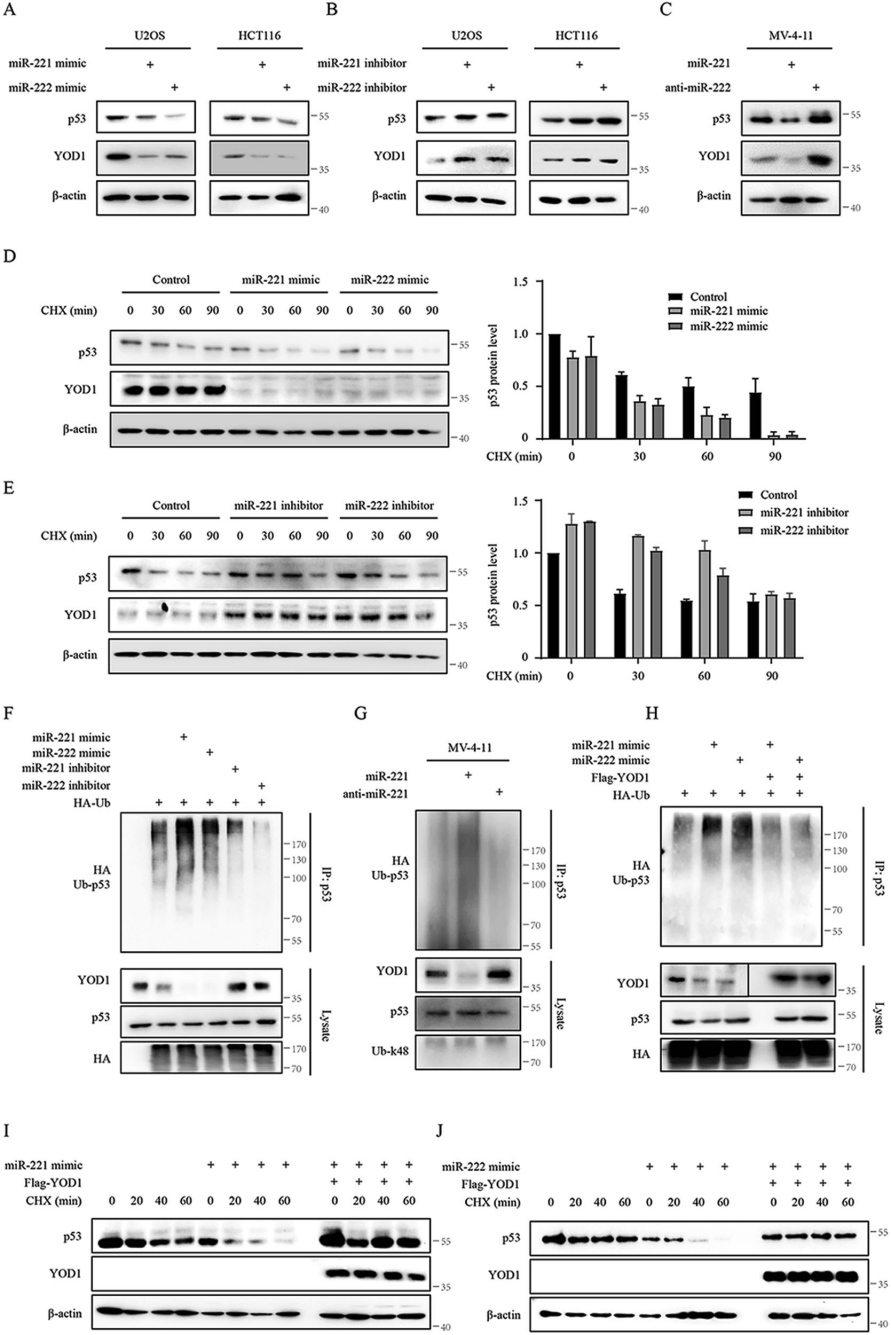
miR-221/222 enhances p53 ubiquitination by downregulating YOD1. **A**, **B** U2OS and HCT116 cells were transfected with miRNA mimic or inhibitors, and expressions of p53, YOD1, and β-actin was detected by IB. **C** MV-4-11 cells were infected with lentiviruses carrying vector control, miR-221, and anti-miR-221 for 96 h. Cell lysates were subjected to IB with specified antibodies. **D**, **E** U2OS cells were transfected with miRNA mimics or inhibitors for 36 h and then treated with 50 μg/ml cycloheximide (CHX) for the indicated time. This was followed by detection of p53, YOD1, and β-actin using IB (left). Relative protein levels of p53 in reference to β-actin were quantified by performing densitometric analysis. **F** U2OS cells were co-transfected with HA-Ub and control, miRNA mimic or miRNA inhibitors for 36 h and then treated with 5 μM MG132 for 4 h. Cell lysates were immunoprecipitated with a p53 antibody and subjected to IB with HA, p53, and YOD1 antibodies. **G** MV-4-11 cells were infected with lentiviruses carrying vector control, miR-221, and anti-miR-221 for 96 h and treated with MG132 for 4 h. Cell lysates were subjected to ubiquitination assays and then immunoblotting with specified antibodies. **H** U2OS cells were co-transfected with HA-Ub, Flag-YOD1 or control and miRNA mimics for 36 h and then treated with MG132. Cell lysates were immunoprecipitated with a p53 antibody and then subjected to immunoblotting with HA, p53, Flag and YOD1 antibodies. **I**, **J** U2OS cells were co-transfected with Flag-YOD1 or miRNA mimics for 36 h and then treated with CHX (50 μg/ml) for the indicated time. This was followed by detection of p53, Flag and β-actin by immunoblotting. Representative results are shown from triplicate experiments with similar results.

The data described above demonstrate that YOD1 stabilizes p53 and is a target of miR-221/222. Presumably, miR-221/222 can downregulate p53. Indeed, this is the case. In both U2OS and HCT116 cells, the protein levels of p53 decreased upon treatment with miR-221/222 mimics and increased upon treatment with miR-221/222 inhibitors, accompanied by a corresponding decrease and increase in the YOD1 protein (Fig. 5A, B). It should be pointed out that these miRNA mimics and inhibitors did not affect p53 at the RNA level (Supplementary Fig. 7 A). Consistent results were also obtained with MV-4–11 AML cells were lentiviral miR-221 infection decreased the expression of YOD1 and p53, whereas lentiviral anti-miR-221 enhanced its expression (Fig. 5C). Treatment of cells with CHX blocked the synthesis of new proteins and allowed us to assess the degradation rate of p53. As shown in Fig. 5D and E, miR-221/222 mimics accelerated degradation, while miR-221/222 inhibitors stabilized the protein level of p53. We further co-transfected cells with HA-Ub, and ubiquitination assays showed that miR-221/222 mimics enhanced p53 ubiquitination, while the miR-221/222 inhibitors reduced it (Fig. 5F and Supplementary Fig. 7B). By infecting MV-4–11 cells with recombinant lentiviruses, we also found that miR-221 enhanced p53 ubiquitination, whereas anti-miR-221 decreased p53 ubiquitination (Fig. 5G). As expected, overexpression of YOD1 attenuated the accelerated degradation and enhanced ubiquitination of p53 caused by miR-221/222 mimics (Fig. 5H-J). Together, these data indicate that miR-221/222 enhances the ubiquitination of p53 and causes its degradation by targeting YOD1.

### Altered expression of YOD1 affects sensitivity of AML cells to FLT3 inhibitors

**Fig. 6:**
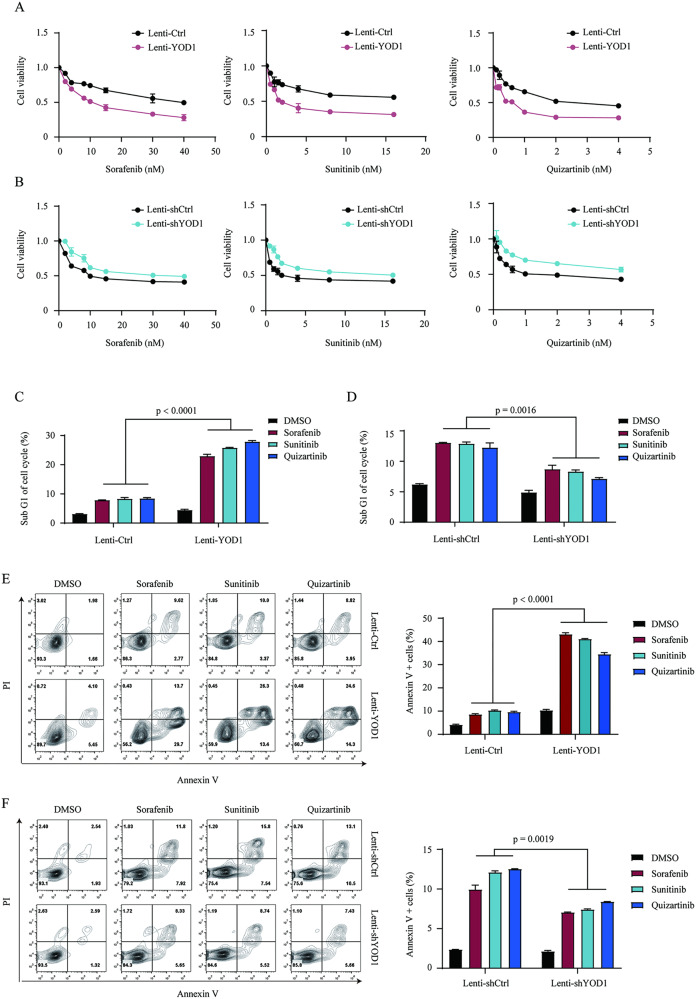
Altered expression of YOD1 affects sensitivity of AML cells to TKIs. MV-4-11 cells were infected with the indicated control or recombinant lentiviruses carrying YOD1 or shYOD1 for 48 h and then treated with puromycin for 24 h. The cells were treated with TKIs for 48 h before further analysis. **A**, **B** CCK8 cell viability assays demonstrated increased and decreased sensitivity of MV-4-11 cells towards TKIs, respectively. **C**, **D** Cell cycle analyses revealed that TKIs induced an increase in G1 upon overexpression of YOD1 and decreased the number of subG1 after YOD1 knockdown. **E**, **F** Annexin V/PI staining revealed that TKIs increased cell death upon overexpression of YOD1 and decreased cell death after YOD1 knockdown. Data represent similar results from triplicate experiments.

**Fig. 7:**
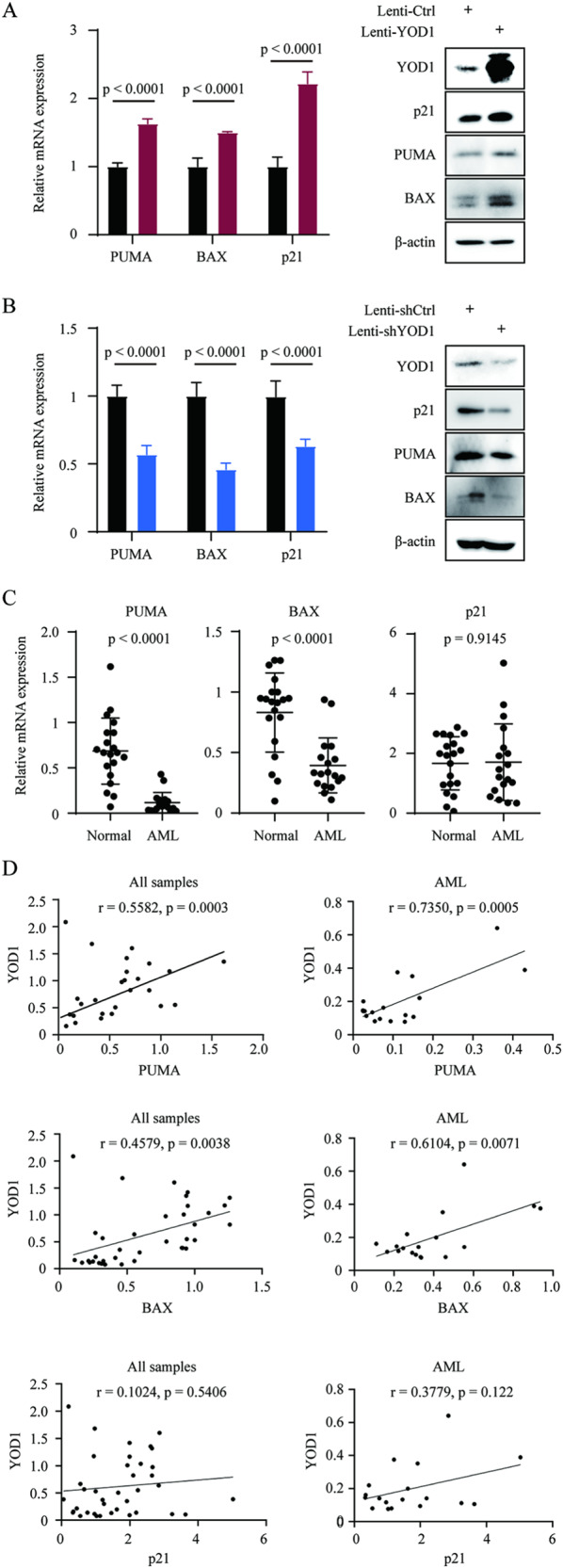
YOD1 expression affects p53 downstream genes. **A**, **B** MV-4-11 cells were infected with the indicated control and recombinant lentiviruses carrying YOD1 or shYOD1 for 48 h and selected with puromycin for 24 h. Expressions of BAX, PUMA, and p21 were analyzed via qPCR with GAPDH as a reference or immunoblotting with indicated antibodies. **C** The relative expression of PUMA, BAX, and p21 in leukocytes from healthy donors and AML patients was analyzed using qPCR. **D** Expression of YOD1 is positively correlated with that of PUMA, BAX, or p21 expression in leukocytes from AML patients and healthy donors or only AML patients. The r values represent Pearson’s correlation coefficients. Representative results are shown from triplicate experiments with similar results.

The protein level of p53 has a central role in controlling cell survival and apoptosis upon drug treatment. We postulated that by regulating p53 ubiquitination, YOD1 may profoundly affect the sensitivity of cells to anticancer drugs. TKIs targeting FLT3 have been found to be effective in treating AML with FLT3 mutations, although drug efficacy needs to be improved and resistance needs to be overcome [38**–**40]. We tested the inhibition of MV-4–11 leukemia cells by TKIs sorafenib, sunitinib, and quizartinib, which have been clinically proven to be effective in FLT3-ITD-posiotive AML. MV-4–11 cells contained FLT3-ITD mutant and wild-type p53. We constructed YOD1 over-expression and YOD1 knockdown lentiviruses using GFP as a separate marker for infection efficiency. After infection with recombinant lentiviruses, MV-4–11 cells were treated with puromycin to enrich the infected cells. As indicated by the flow cytometric analysis of GFP-positive cells, we achieved a lentivirus infection efficiency of > 90% (Supplementary Fig. 8A). Importantly, this altered expression of YOD1 had profound effects on the response of MV-4–11 cells toward TKIs, as indicated by CCK8 cell viability assays (Fig. 6A, B), cell cycle analyses (Fig. 6C, D), and apoptotic cell staining (Fig. 6E, F). Similar results were obtained with MOLM13 leukemia cells (Supplementary Fig. 8B and C). Taken together, our data demonstrated that the inhibitory effects of TKIs on cells were enhanced by YOD1 overexpression and reduced by YOD1 knockdown. We then analyzed the expression of p53 downstream genes in YOD1 over-expression and knockdown cells. As expected, qPCR analysis demonstrated that the mRNA levels of p53 downstream genes, including PUMA, BAX, and p21, were increased in YOD1-overexpressing cells and were reduced in YOD1 knockdown cells (Fig. 7A, B). We further assessed the expression of PUMA, BAX, and p21 in leukocytes from patients with AML and healthy donors. The data indicated that the expression of PUMA and BAX was significantly suppressed in AML patients, although the reduction in p21 expression was not statistically significant (Fig. 7C). Interestingly, the expression levels of YOD1 are positively correlated with expressions of p21, BAX, and PUMA with r values of 0.21 to 0.65 and *P* values of <0.05 (Fig. 7D). Together, our data suggest that the survival of AML cells is facilitated by reduced expression of YOD1, leading to instability of p53 and decreased expression of pro-apoptotic genes.

## Discussion

The stability of p53 is closely related to the pathogenesis of various cancers and the response of cancer cells to drug treatments [41, 42]. Numerous studies have demonstrated that the E3 ligase MDM2 is often deregulated in cancer cells and plays a key role in controlling the protein level of p53 via ubiquitination. Consequently, MDM2 inhibitors have been extensively explored as anti-cancer therapies [43, 44]. However, the toxicity of these inhibitors to normal cells in the hematopoietic and gastrointestinal systems is a major problem [2]. Another hurdle is the development of drug resistance due to mechanisms such as overexpression of MDM4 and the occurrence of gene mutations that disrupt the binding of MDM2 inhibitors [45, 46]. Although no MDM2 inhibitors have been approved for cancer treatment, enhancing the stability of p53 remains a promising approach in anticancer drug development [46**–**48]. We believe that it is necessary to explore other mechanisms underlying the regulation of p53 stability.

Our present study suggests a critical role for the deubiquitination enzyme YOD1 in the control of p53 stability, and thus provides new potential drug targets. The entire DUB family contains seven subfamilies, and more than ten DUBs have been reported to target the p53 pathway [49]. USP7 has been studied for its ability to regulate the stability of p53 and MDM2 [28], and USP7 inhibitors have been developed for cancer therapy [50, 51]. Our study revealed that YOD1 expression is altered in AML cells and is a major DUB responsible for deubiquitination and stability of p53. YOD1 first received attention as a component of the multi-protein complex with p97 as the core, which is involved in the dislocation of misfolded proteins in the endoplasmic reticulum [52]. Studies have shown that the DUB activity of YOD1 contributes to the control of antigen-specific CD8^+^T cell responses during the immune process [53]. DUBs are ubiquitin chain linkage-specific, and YOD1 cleaves various types of ubiquitination bonds and preferentially targets long-chain ubiquitination in vitro [36]. Our data demonstrated that YOD1 specifically cleaves the K48 ubiquitin chain to stabilize p53 protein in a catalytic activity-dependent manner. We believe that reagents that increase the expression or activation of YOD1 may have a potential role in the treatment of AML.

Our study also demonstrated a role for miR-221/222 in the downregulation of YOD1 expression and, consequently, the stability of p53. miRNAs are small single-stranded non-coding RNA molecules that base pair with complementary sequences in mRNA molecules that cause RNA silencing [54]. Its specific expression is often considered a biomarker for some diseases. miRNAs are involved in almost all aspects of AML pathogenesis and development, including cell proliferation, survival, and differentiation [55**–**57]. Our study provided further evidence that miR221/222 are overexpressed in AML, which is consistent with previous reports [37, 58]. More importantly, our data demonstrated that high expression of miR-221/222 is responsible for decreased YOD1 expression in AML. It is conceivable that miR-221/222 may serve as an unfavorable prognostic marker for AML. Indeed, a recent meta-analysis showed that high expression of miR-221/222 in various types of cancers is generally associated with poor overall survival [59]. miRNA mimics and inhibitors (anti-miRNAs) have shown promise in preclinical studies for the treatment of cancer and other diseases. Several miRNA-targeted therapies have been tested in clinical trials. These include phase I clinical trials of tumor suppressor miRNA miR-34 mimics for cancer treatment and phase II clinical trials of miRNA inhibitors targeting onco-miRNAs miR-122 for the treatment of hepatitis [60, 61]. MiR-221/222 are two of the most significantly upregulated miRNAs in hepatocellular carcinoma and have been reported to target tumor suppressor genes, including p27KIP1, PTEN, and TIMP3 [62, 63]. In fact, a preclinical study demonstrated that cholesterol-modified anti-miR-221 reduced tumor size in mice and prolonged their survival [64]. Considering our data, anti-miR-221/222 could be used to treat AML by increasing YOD1 expression, thereby stabilizing p53. The miRNA-based approach has exciting therapeutic potential because miRNAs are endogenous molecules that often inhibit multiple targets; thus, the possibility of developing resistance through target site mutations is unlikely [65]. Two major issues to be addressed are the maintenance of miRNA stability in vivo and the improvement of the delivery system.

Mutations in FLT3 occur in ~30% of AML cases and are associated with disease progression, an increased risk of recurrence, and shorter overall survival [66, 67]. Mutant FLT3 leads to continuous activation of downstream signaling pathways, and FLT3 inhibitors have been found to be highly effective in treating AML, although drug resistance often develops [68, 69]. Several studies have reported the efficacy of FLT3 inhibitors combined with MDM2 inhibitors in the treatment of AML [70, 71], suggesting stabilizing p53 leads to more effective treatment. Importantly, our data demonstrate that the sensitivity of FLT3-ITD-bearing AML cells toward FLT3 inhibitors is affected by the expression of YOD1. The effectiveness of FLT3 inhibitors was significantly enhanced when YOD1 was overexpressed and inhibited when it was suppressed. We believe that stabilization p53 of by deubiquitination is a critical factor in the treatment of AML. We postulate that miR-221/222 inhibitors can be used to increase the level of YOD1 and stabilize p53, thereby sensitizing AML cells toward FLT3 inhibitors and overcoming drug resistance. However, it is interesting to note that a recent study demonstrated the stabilizing effects of YOD1 on oncogenic PML/RARα frequently found in promyelocytic leukemia (APL) [72]. In this regard, inhibition rather than activation of YOD1 would be therapeutically beneficial.

## Methods

### Blood and bone marrow specimens

Normal and AML peripheral blood samples and normal bone marrow samples were collected from the Seventh Affiliated Hospital of Sun Yat-sen University, under an approved institutional review board protocol. Whole blood leukocytes were isolated using an erythrocyte lysis buffer (Abcam, Cambridge, UK). FAB classifications and gene mutations for AML patients are provided in Supplementary Table 1.

### Cell culture and transfection

U2OS, MOLM13, and MV-4–11 cells were cultured in RPMI medium (Gibco, Carlsbad, CA, USA) supplemented with 10% fetal bovine serum (FBS) (Hyclone, Logan, UT, USA). HCT116 and 293 T cells were cultured in DMEM medium (Gibco) supplemented with 10% FBS. Cultured cells were transfected with plasmid DNAs, miRNA mimics, miRNA inhibitors, or siRNAs using Lipofectamine 3000 reagent (Invitrogen, Carlsbad, CA, USA).

### cDNA constructs, siRNA, and miRNA mimics/inhibitors

Full-length human wild-type (WT) YOD1 cDNA was cloned into the pFlag-CMV2 vector (Sigma-Aldrich, St Louis, MO). Full-length human wild-type p53 was cloned into the pCMV-Myc-N vector (Clontech, Mountainview, CA, USA), whereas full-length human wild-type and various mutant forms of ubiquitin were cloned into the pCMV-HA-N vector (Clontech, Mountain View, CA). Point and domain-truncation mutants of YOD1 and p53 were made through site-directed mutagenesis in the original vectors. The primers used are shown in Supplementary Table 2. YOD1 siRNA was purchased from Santa Cruz Biotechnology (Dallas, TX, USA). The miR-221/222 mimics and inhibitors were obtained from Sigma-Aldrich.

### Lentiviral constructs

A lentivirus packaging system was purchased from Hanbio (Shanghai, China). The system contained two helper plasmids (pSPAX2 and pMD2G) and a shuttle plasmid LV011-pHBLV-CMV-MCS-3flag-EF1-T2A-Zsgreen-Puro (LV011) for gene overexpression or pLKO.1-U6-EF1a-copGFP-T2A-puro (pLKO.1) for gene knockdown. The shRNA sequence was CCGGGCACTGGAATTAGCAGATGAAC-TCGAGTTCATCTGCTAATTCCAGTGCTTTTTGAATT. The lentiviral miR-221 and anti-miR-221 sequence was CCGGA AATCTACATTGTATGCC- AGGTCTCGAGACCTGGCATACAATGTAGATTTTTTTTGAATT and CCGGACCTGGCATACAATGTAGATTTCTCGAGAAATCTACATTGTATG CCAGGTTTTTTGAATT.

### Quantitative reverse-transcription PCR

Total RNA was extracted using the MiniBEST Universal RNA Extraction Kit (TaKaRa, Shiga, Japan), and reverse transcription with equal amounts of RNAs was performed using the PrimeScript RT-PCR Kit (TaKaRa, Shiga, Japan). qPCR was conducted using SYBR Green I (Vazyme, Nanjing, China) as the fluorescent dye and analyzed using Precision Melt Analysis software (Bio-Rad, Carlsbad, USA). The primers listed in Supplementary Table 3.

### Stem-loop RT-qPCR

The miR-221/222-3p sequences were based on miRBase (http://www.mirbase.org/). Primers were designed according to the method described by Varkonyi-Gasic and Hellens [73]. The specificity of a stem-loop RT primer for an individual miRNA is conferred by a six-nucleotide extension at the 3'-end. The products obtained from the stem-loop RT-PCR were detected by qPCR using forward (specific) primers and reverse (universal) primers. The primers listed in Supplementary Table 4.

### Immunoblotting

Immunoblotting was performed as described [74, 75]. The antibodies used were anti-Flag (Sigma-Aldrich), polyclonal anti-YOD1 (Proteintech, Rosemont, IL), monoclonal and polyclonal anti-p53 (Cell Signaling Technology, Beverly, MA and Santa Cruz Biotechnology respectively), anti-Myc, anti-PUMA, anti-BAX, and anti-MDM2 antibodies (Santa Cruz Biotechnology), anti-K48 ubiquitin and β-actin (Cell Signaling Technology), anti-HA (Abcam), and horseradish peroxidase-conjugated secondary antibodies (Cell Signaling Technology).

### Deubiquitination assays

U2OS and HCT116 cells were transfected with vectors, as indicated. MOLM13 and MV-4-11 cells were infected with recombinant lentivirus, as previously described [74].

### Immunostaining

Immunostaining was performed as described [76, 77]. The cells were fixed with 4% polymethanol. After permeation with 0.3% Triton X-100. Thereafter, incubated with p53 and YOD1 antibodies overnight. Secondary antibodies labeled with Alexa Fluor^TM^ Plus 488 and 555 (Invitrogen) and staining with DAPI. Images were captured under a Zeiss LSM 510 confocal laser scanning microscope.

### Cell viability, apoptosis, and cell cycle assays

For cell viability assays was measured using the cell counting Kit-8 (CCK-8) method. For apoptosis analysis, cells were stained with APC-Annexin V (BD Biosciences, San Jose, USA) and propidium iodide. To assess cell cycle arrest, cells were fixed with ethanol overnight and stained with propidium iodide in the presence of RNase A. A CytoFlex LX Flow Cytometer (Beckman Coulter, USA, CA) was used according to the manufacturer’s instructions. FlowJo (V10, FlowJo, Treestar, OR, USA) was used for apoptosis and cell cycle analyses.

### Luciferase activity assays

Luciferase constructs were made using the pmirGLO Dual-Luciferase miRNA Target Expression Vector System (Promega, Madison, USA). A YOD1 3′ UTR fragment was generated by RT-PCR from MV-4-11 cell total RNA. The primer sequences are provided in Supplementary Table 5. Luminescence was measured using a dual-luciferase reporter assay system (Promega).

### Statistical analysis

Optical intensity was measured using the AlphaEasy program (version 5.1; Alpha Innotech, San Leandro, CA, USA). Data were analyzed using GraphPad Prism version 8.0.2 (GraphPad Software Inc., San Diego, CA). All numerical values are presented as the mean ± SD. Difference between two groups was calculated using unpaired student t-test. Differences among multiple groups were calculated using two-way ANOVA. *p* < 0.05 was considered significant.

## Supplementary information

Supplementary info

Original Data File

Certificate of editing

## Data Availability

All data are ready to be shared with researchers of interest (please contact joe-zhao@ouhsc.edu and/or cheny653@mail.sysu.edu.cn).
