# Supplementary material for: miR-221/222 induce instability of p53 By downregulating deubiquitinase YOD1 in acute myeloid leukemia
Source: Cell Death Discov. 2023 Jul 15;9:249. doi: 10.1038/s41420-023-01537-4 (PMC10349814; doi:10.1038/s41420-023-01537-4)
Supplement: Supplementary file 1 — Supplementary info [file 41420_2023_1537_MOESM1_ESM.docx]

**SUPPLEMENTARY INFORMATION**

**miR-221/222 Induce Instability of p53 By Downregulating Deubiquitinase YOD1 in Acute Myeloid Leukemia**

Han Zhong Pei^1,#^, Zhiyong Peng^2,#^, Xiaomei Zhuang^1,#^, Xiaobo Wang^1^, Bo Lu^1^, Yao Guo^1^, Yuming Zhao^1^, Dengyang Zhang^1^, Liuting Yu^1^, Chunxiao He^1^, Suk-Hwan Baek^3*^, Zhizhuang Joe Zhao^4*^, Xiaojun Xu^1*^ and Yun Chen^1*^

^1^Department of Hematology, The Seventh Affiliated Hospital, Sun Yat-sen University, Shenzhen, 518107, Guangdong, China. ^2^Nanfang-Chunfu Children's Institute of Hematology, Taixin Hospital, Dongguan, Guangdong, China. ^3^Department of Biochemistry & Molecular Biology, College of Medicine, Yeungnam University, 170 Hyeonchung-ro, Nam-gu, Daegu 42415, South Korea. ^4^Department of Pathology, University of Oklahoma Health Sciences Center, 940 Stanton L. Young Blvd., BMSB 451, Oklahoma City, OK 73104 USA.
***Correspondence:** Dr. Yun Chen ([cheny653@mail.sysu.edu.cn](mailto:yun.c0415@hotmail.com)), Edmond H. Fischer Translational Medical Research Laboratory, Precision Medicine Center, The Seventh Affiliated Hospital, Sun Yat-sen University, Shenzhen, 518107, Guangdong, China; Dr. Xiaojun Xu (xuxj29@mail.sysu.edu.cn), Department of Hematology, The Seventh Affiliated Hospital, Sun Yat-sen University, Shenzhen, 518107, Guangdong, China; Dr. Zhizhuang Joe Zhao ([joe-zhao@ouhsc.edu](mailto:joe-zhao@ouhsc.edu)), Department of Pathology, University of Oklahoma Health Sciences Center, 940 Stanton L. Young Blvd., BMSB 451, Oklahoma City, OK 73104 USA; Dr. Suk-Hwan Baek ([sbaek@ynu.ac.kr](mailto:sbaek@ynu.ac.kr)), Department of Biochemistry & Molecular Biology, College of Medicine, Yeungnam University, 170 Hyeonchung-ro, Nam-gu, Daegu 42415, South Korea.

**Supplementary figure and figure legends**

**

**

**Supplemental Figure 1: Correlation analysis of deubiquitinase expressions and myeloblast percentages in AML.** Peripheral blood leukocytes were isolated by centrifugation after red blood cell lysis from AML patients (n=18). Relative expression levels of DUBs in reference to GAPDH were determined by qPCR using the 2ΔΔCT method.





**Supplementary Figure 2: There is no significant change in p53 mRNA levels in AML patient blood cells and U2OS2 cells with altered YOD1 expression.** (A) Relative expressions of p53 in AML patients and healthy donors were analyzed by qPCR. (B, C) U2OS cells were transfected with indicated cDNA constructs or siRNA for 36 h. Expressions of p53 were analyzed by performing qPCR. P>0.05 (ns). Data = mean ± SD (n≥3).


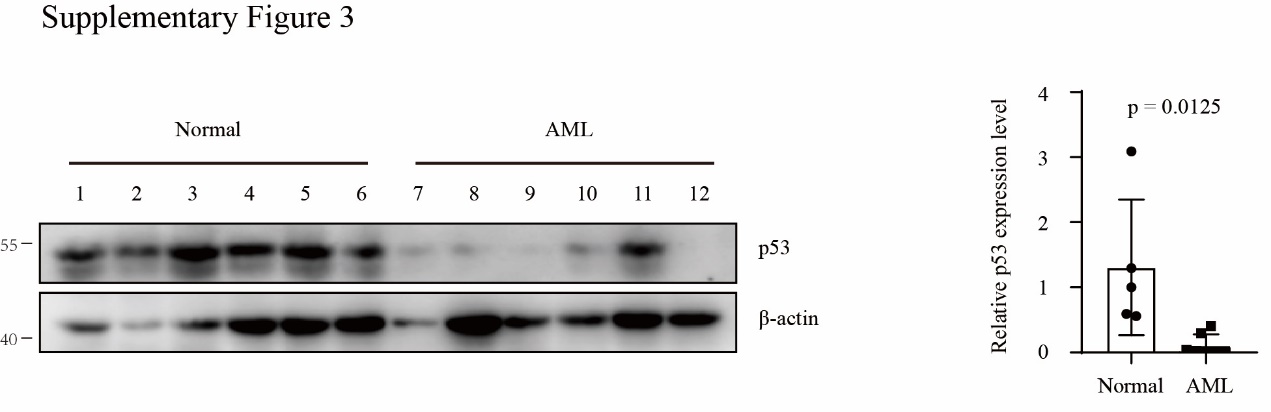


**Supplemental Figure 3: Protein levels of p53 are reduced in AML peripheral leukocytes patients.** healthy (n = 6) and AML (n = 6) peripheral leukocytes cell lysates were subjected to immunoblotting with p53 and β-actin. Relative protein levels of p53 were determined by densitometric analyses with β-actin as a reference.

**
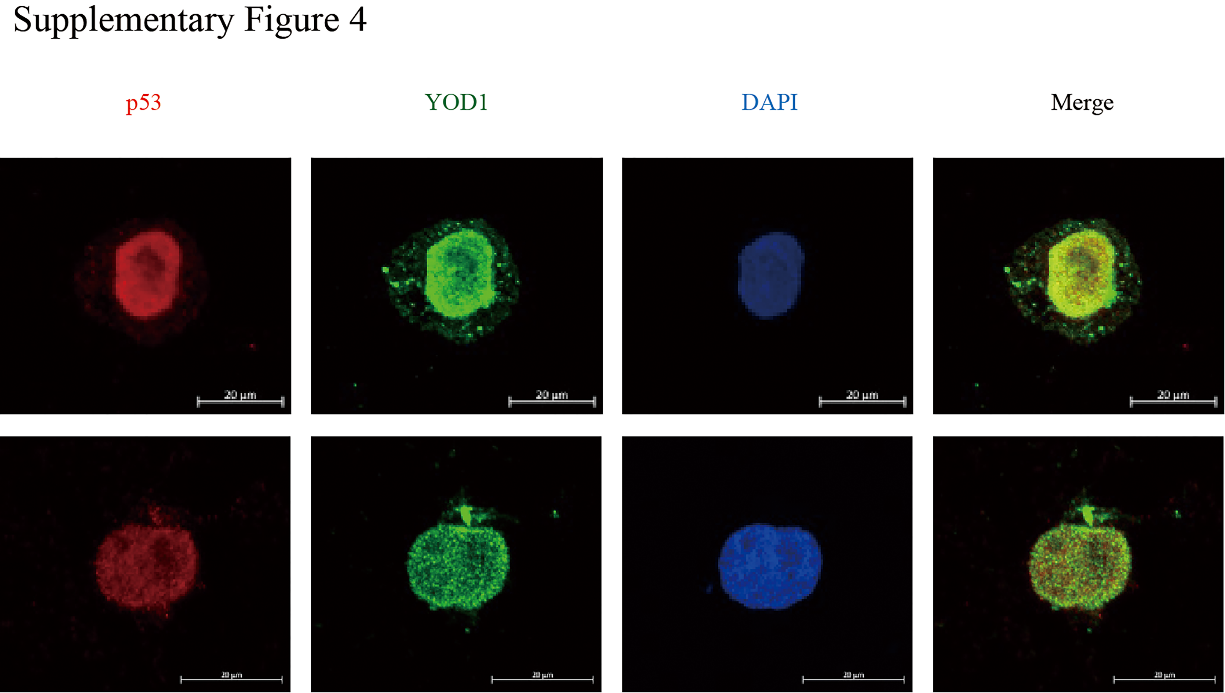
**

**Supplemental Figure 4:** **p53 and YOD1 are colocalized in MV-4-11 cells.** Colocalization of p53 and YOD1 revealed by indirect immunofluorescent staining in MV-4-11 cells. Bar 20μm.


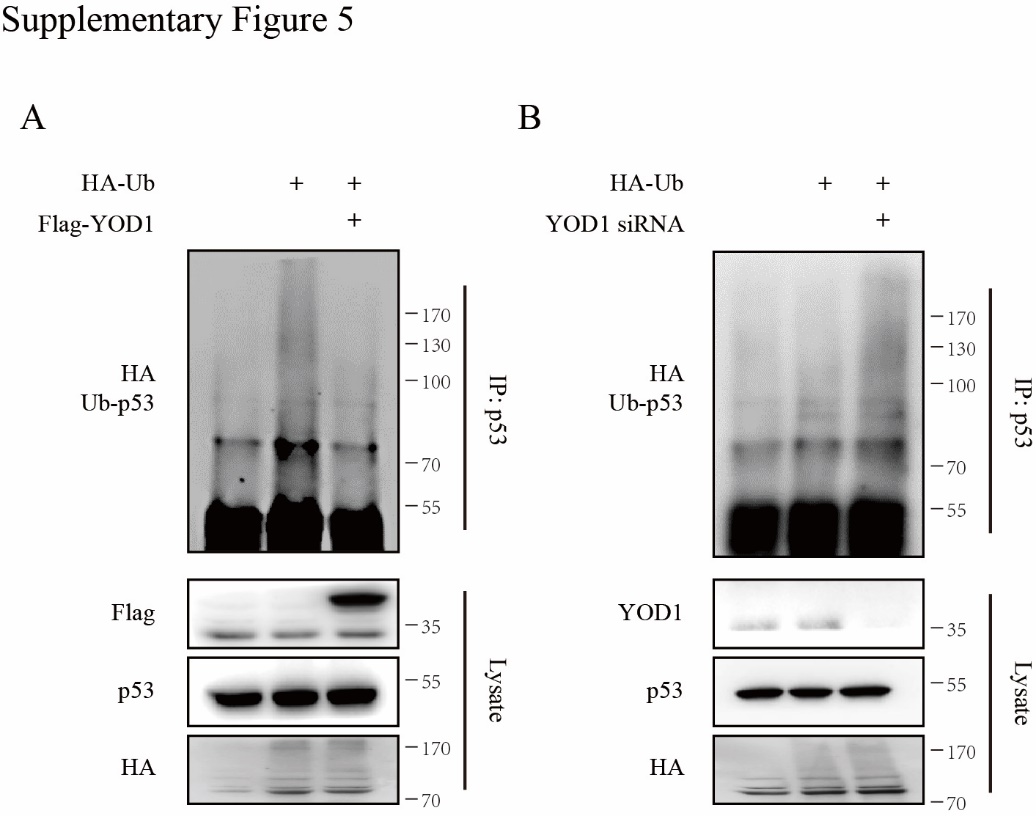


**Supplementary Figure 5: Altered expressions of YOD1 affect p53 ubiquitination in HCT116 cells.** HCT116 cells were transfected with indicated cDNA constructs (A) or siRNA (B) for 36 h and then treated with 5 μM MG132 for 4 h. Cell extracts were immunoprecipitated with a p53 antibody and then subjected to immunoblotting with HA, p53 and Flag antibodies. Representative data from 3 experiments with similar results are shown.


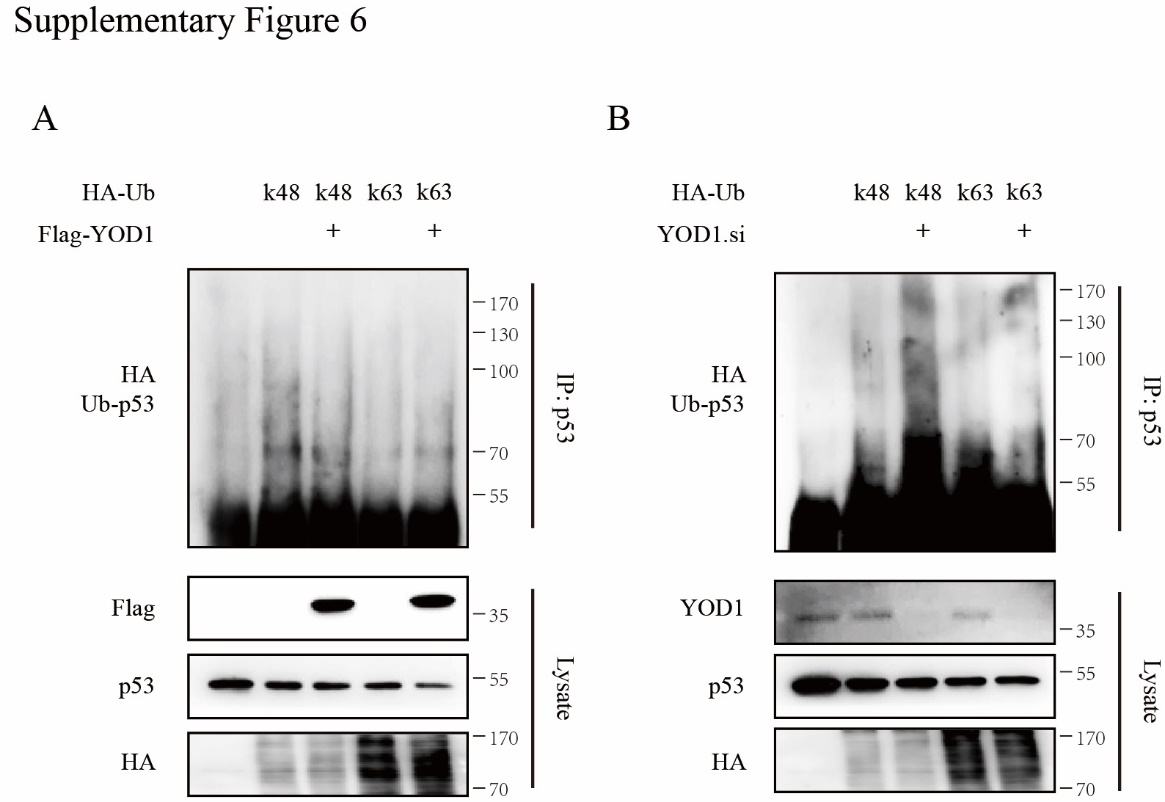


**Supplementary Figure 6: YOD1 overexpression and knockdown affect K48- but not K63-linked ubiquitination of p53.** U2OS cells were co-transfected with HA-ubiquitin (wild type and K48 or K63 mutant) and Flag-YOD1 plasmid (A) or YOD1 siRNA (B) as indicated. Cells were treated with 5 μM MG132 for 4 h before extraction for immunoprecipitation and immunoblotting with indicated antibodies. Data represent r3 independent experiments with similar results.


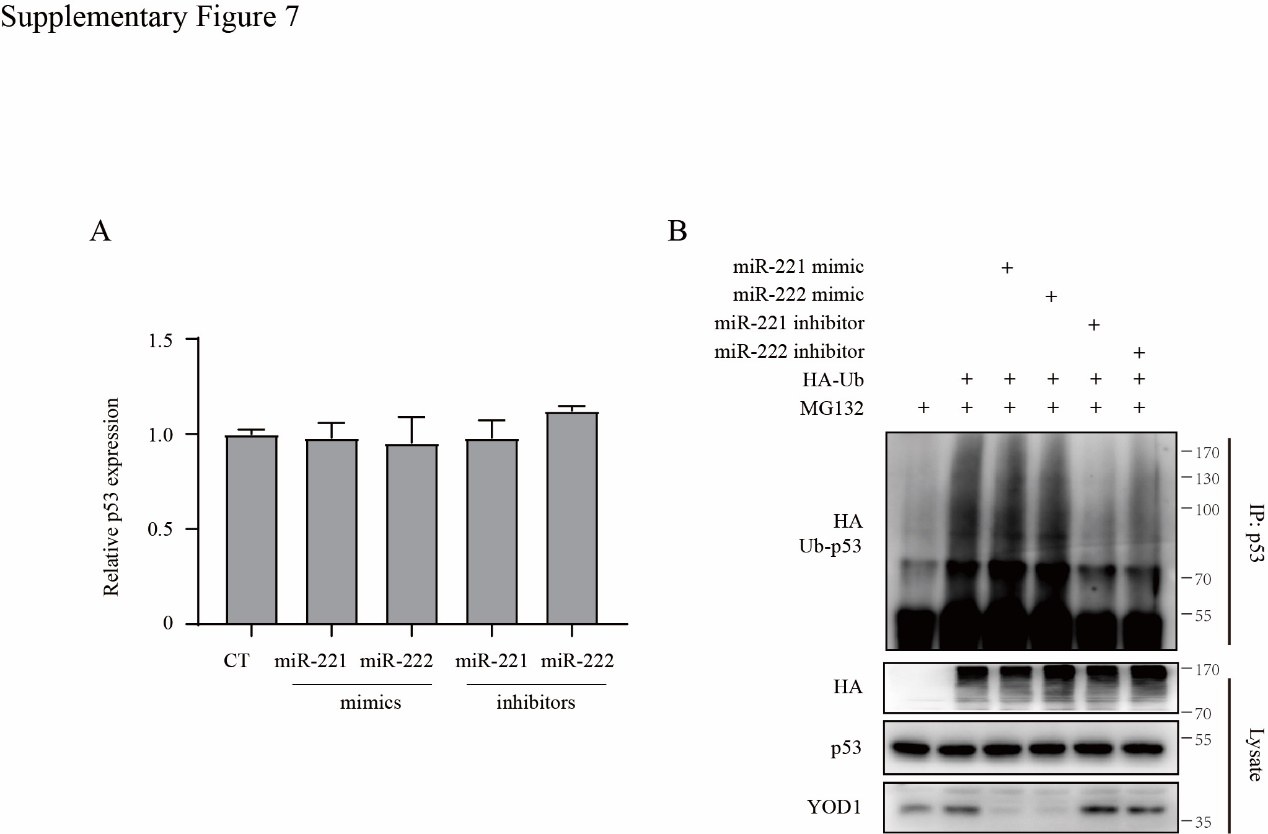


**Supplementary Figure 7: Transfection of U2OS and HCT116 with miR-221/222.** (A) U2OS cells were transfected with miR-221-3p and miR-222-3p inhibitors or mimics as indicated. mRNA expressions of p53 were analyzed by qPCR. (B) HCT116 cells were co-transfected with HA-Ub and miRNA mimics or inhibitors for 36 h and then treated with 5 μM MG132 for 4 h. Cell extracts were immunoprecipitated with a p53 antibody and then subjected to immunoblotting with HA, p53 and YOD1 antibodies. Data represent similar results from triplicate experiments.


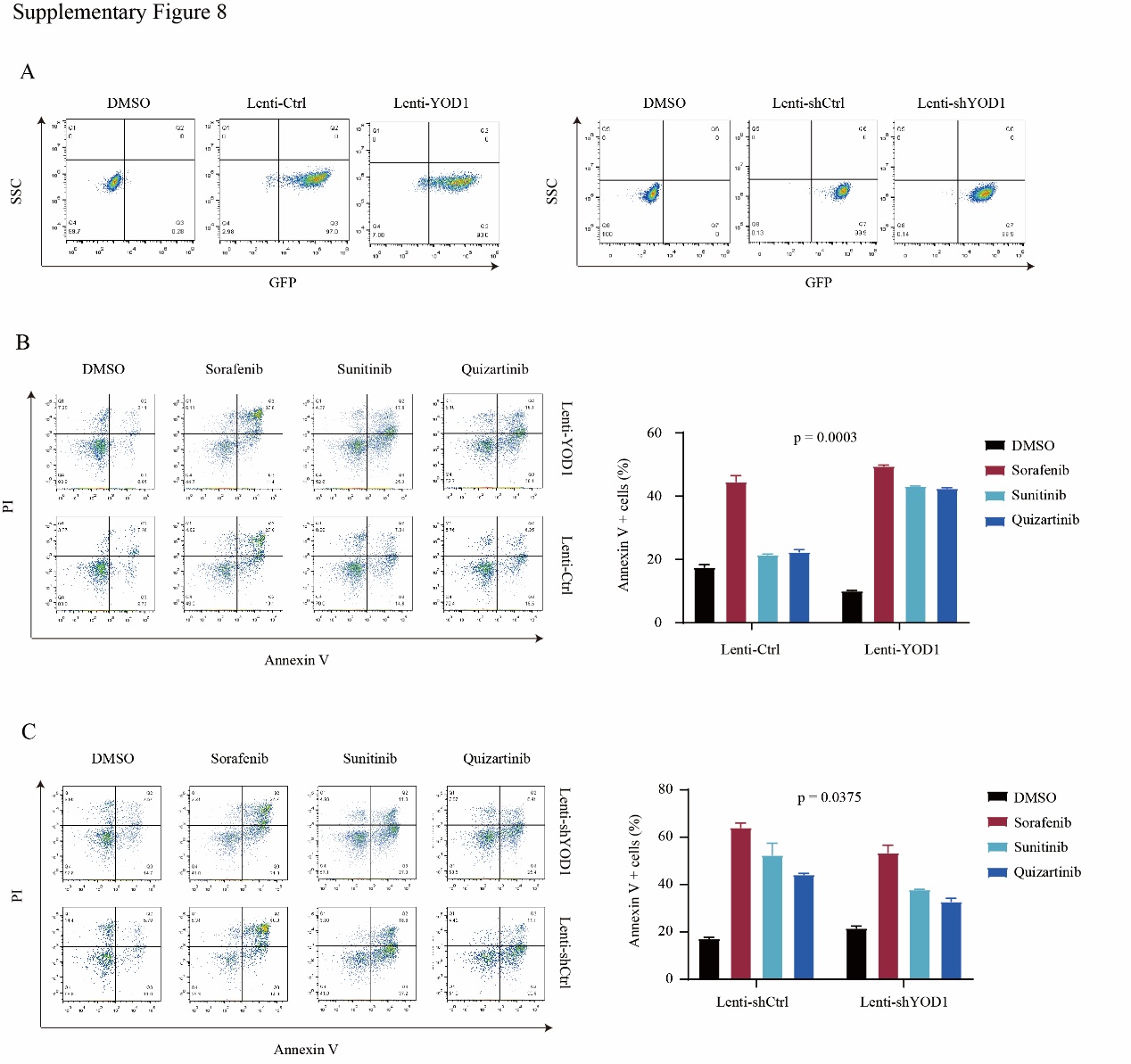


**Supplementary Figure 8: Altered expression of YOD1 affects apoptosis of AML cells induced by TKIs.** (A) MV-4-11 cells were infected with indicated control and recombinant lentiviruses carrying YOD1 or shYOD1. The expression of GFP was detected by flow cytometry. (B, C) MOLM13 Cells were infected with indicated control and recombinant lentiviruses carrying YOD1 or shYOD1 and then treated with indicated TKIs for 24 h. Cells were stained with APC- Annexin V and PI and then subjected to flow cytometry analysis. Shown are representative data from 3 independent experiments with similar results.

**Supplementary Table 1. FAB and gene mutation classifications of AML patients**

| Patients | p53 Mutation | FAB category | Class I Mutations (Confer Proliferation & Survival) | Class II Mutations (Confer Differentiation & Apoptosis) | Class III Mutations (Confer Epigenetic Alteration) |
| --- | --- | --- | --- | --- | --- |
| AML1 | None | M6 | NRAS | None | None |
| AML2 | None | M5 | None | NPM1 | IDH2 |
| AML3 | None | M2 | None | None | None |
| AML4 | None | M1 | FLT3-ITD | NPM1 | IDH2 |
| AML5 | None | M2 | KRAS, NRAS | PTEN | None |
| AML6 | None | M0 | None | CEBPA | None |
| AML7 | None | M5 | FLT3-ITD | RUNX1 | IDH2 |
| AML8 | None | M5 | FLT3-ITD | None | None |
| AML9 | None | M5 | None | None | None |
| AML10 | None | M3 | None | None | None |
| AML11 | N/A | M5 | N/A | N/A | N/A |
| AML12 | None | M5 | FLT3-ITD, NRAS | CEBPA | None |
| AML13 | None | M2 | None | CEBPA, WT1 | None |
| AML14 | N/A | M4 | N/A | N/A | N/A |
| AML15 | None | M5 | C-KIT | None | None |
| AML16 | None | M2 | None | RUNX1 | ASXL1 |
| AML17 | N/A | M3 | N/A | N/A | N/A |
| AML18 | None | M2 | None | None | None |

**Supplementary Table 2. Site-directed mutagenesis primers for YOD1 and p53**

| Flag-YOD1 F | GAATTCGATGTTTGGCCCCGCT |
| --- | --- |
| Flag-YOD1 R | GGATCCTCACACTTCTCCAAAGTTGGTA |
| YOD1 C160S F | CTCTTCCCTCTTTACTAGTGTGTACTATG |
| YOD1 C160S R | GAGGGAAGAGTTGTCTGCTGGGAC |
| Myc-p53 F | GAGGCCCGAATTCCGCCTGAAAACAACGTT |
| Myc-p53 R | CTCGAGAGATCTTCACTTGCGGAGATTCTC |
| Myc-p53 △N R | CTCGAGAGATCTTCACTTGCGGAGATTCTC |
| Myc-p53 △M F | GAGGCCCGAATTCCGCCTGAAAACAACGTT |
| Myc-p53 △C F | GAGGCCCGAATTCCGACCTACCAGGGCAGC |
| Flag-YOD1 F | GCGGCCGCGAATTCGATGTTTGGCCCCGCT |
| Flag-YOD1 R | CACCCGGGATCCTCACACTTCTCCAAAGTT |
| Flag-YOD1 △UBX F | GCGGCCGCGAATTCGACCAGAACCGTGGTC |
| Flag-YOD1 △Znf R | CACCCGGGATCCTCAGTTACGCTGAAGTGG |

**Supplementary Table 3. List of primers for RT-qPCR**

| YOD1 F | AGCAAAGTCTTTTACAGTGGAGG |
| --- | --- |
| YOD1 R | CGGTGATGGCGGCAATTT |
| USP3 F | CGGTTTCAACGGTGTTTCCC |
| USP3 R | TGCCTCCGAATATAGCCGTG |
| OTUD5 F | GCCTGCCATCATTCAAACCA |
| OTUD5 R | CCACTGCAGGTAGGATTCCC |
| OTUB1 F | CGGCAACTGTTTCTATCGGG |
| OTUB1 R | AATTCAGTGAAGCCCTGGGA |
| OTUD1 F | GAGAGTCCCACGGTGTCTAC |
| OTUD1 R | GTTCTTCGTCGCGTTTCCTT |
| USP10 F | GGCGGCCATTACACTACAGA |
| USP10 R | CCACTCGGCGGTAATACAGG |
| USP42 F | GGGTGCTGTGTCTTCATTGA |
| USP42 R | AGGGCTTGATCCTTTTGTGG |
| CYLD F | CCTGGGAACTCACATGGTCT |
| CYLD R | TTCATTCAGTCCTGGTGGCT |
| USP49 F | CCTGAACGCTATCACTGCAT |
| USP49 R | AGCGTAGATTCTCCCTTCCA |
| ATXN3 F | ATCAGAGGCTCAGGATCGAT |
| ATXN3 R | TGGACCCGTCAAGAGAGAAT |
| USP11 F | CCTGGTCAGCTGGTATGGTC |
| USP11 R | CCGGACAAGCAGCAGTTCTA |
| USP24 F | GCATGCGGGAATCTGTGTTC |
| USP24 R | ATCAGCAACACAGGGCAAGT |
| OTUD3 F | CCTTTGTGGCAGATTCGTGG |
| OTUD3 R | TCGTAGTGCTCTCCATACCG |
| USP29 F | AGACTGTGGAGATGCAAGCC |
| USP29 R | GGTCCATCAGTGAGTCAGCC |
| USP5 F | CCTCACTTCTCCTCTCCGAC |
| USP5 R | GTAGTAGACAGCTTTGCGGC |
| USP9X F | TGCTCGTACACAGTGGTCAA |
| USP9X R | ACCAGCGATTTCTCTCACCA |
| BAX F | AGCAAACTGGTGCTCAAGGC |
| BAX R | CCACAAAGATGGTCACGGT |
| PUMA F | ACGACCTCAACGCACAGTACG |
| PUMA R | TCCCATGATGAGATTGTACAGGAC |
| p21 F | GGACCTGTCACTGTCTTGT |
| p21 R | ATCTGTCATGCTGGTCTGCC |
| MDM2 F | ACGACAAAGAAAACGCCACA |
| MDM2 R | CGATGGCGTCCCTGTAGA |
| p53 F | GCCTGAGGTTGGCTCTGA |
| p53 R | GTGGTGAGGCTCCCCTTT |
| GAPDH F | AGGGCTGCTTTTAACTCTGGTAA |
| GAPDH R | TGGGTGGAATCATATTGGAACAT |

**Supplementary Table 4. List of primers for Stem-loop RT-PCR primer**

| miR-221-3p stem-loop RT-PCR primer | GTCGTATCCAGTGCAGGGTCCGAGGTATTCGCACTGGATACGACGAAACCCA |
| --- | --- |
| miR-221-3p specific F | AGCTACATTGTCTGC |
| miR-222-3p stem-loop RT-PCR primer | GTCGTATCCAGTGCAGGGTCCGAGGTATTCGCACTGGATACGACACCCAGTA |
| miR-222-3p specific F | AGCTACATCTGGC |
| universal R | GTGCAGGGTCCGAGGT |

**Supplementary Table 5. List of primer used for making luciferase constructs**

| 3U partial WT F | ATGTAGCGCGGCCGCTTCGAGC |
| --- | --- |
| 3U partial MT F | ACGTAACGCGGCCGCTTCGAGC |
| pmirGLO F | GCGGCCGCTTCGAGC |
| pmirGLO R | CTCGAGGCTAGCGAGCTC |
| YOD1 3U F | ACGAGCTCGCTAGCCTCGAGATTGATGTTGAATTGTAGGGTGCTG |
| YOD1 3U R | TGTCTGCTCGAAGCGGCCGCGCCTATACCAGTTTATAAAGTAGACTCATG |
