# Supplementary material for: miR-221/222 induce instability of p53 By downregulating deubiquitinase YOD1 in acute myeloid leukemia
Source: Cell Death Discov. 2023 Jul 15;9:249. doi: 10.1038/s41420-023-01537-4 (PMC10349814; doi:10.1038/s41420-023-01537-4)

Figure 2A:

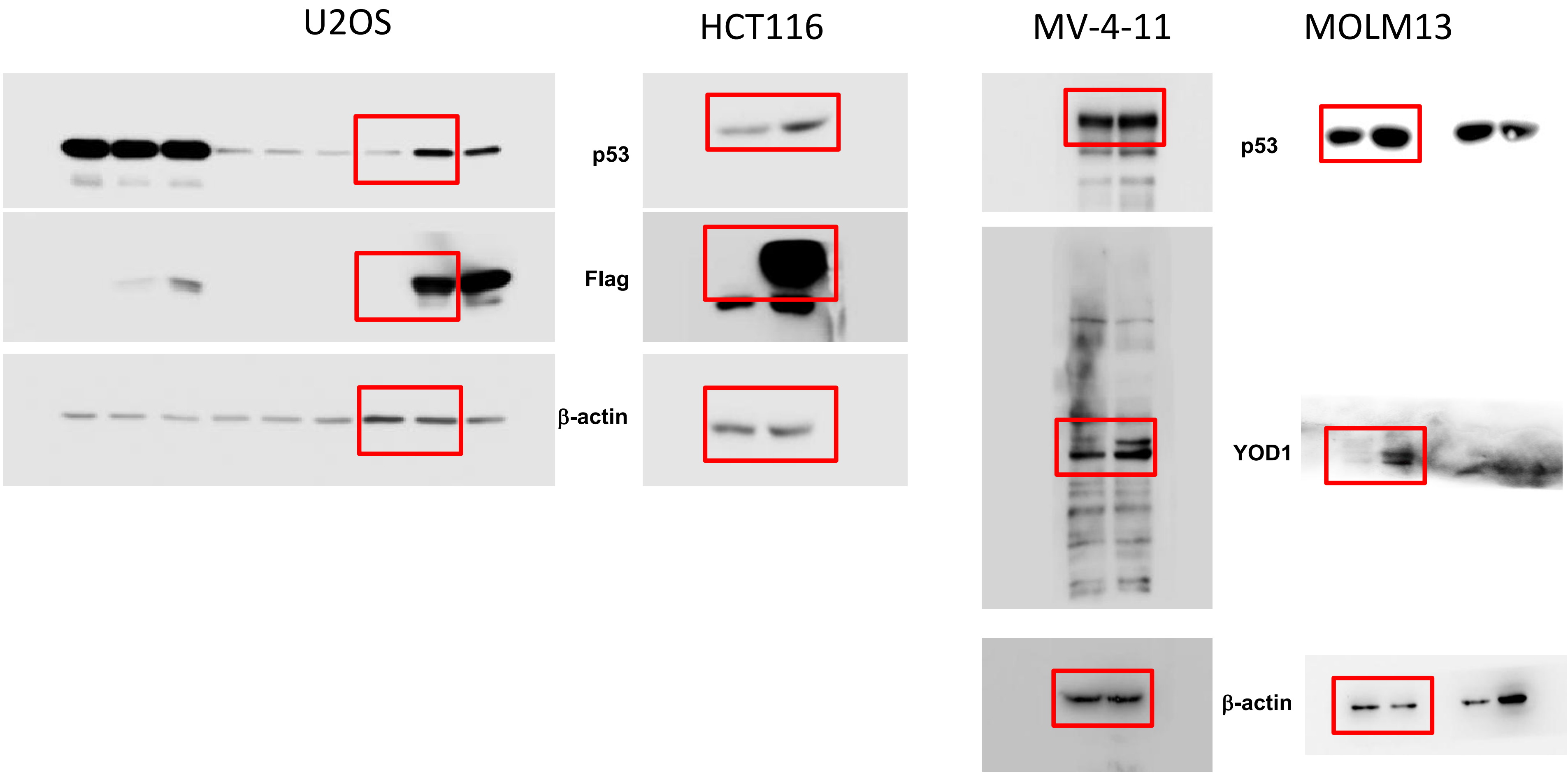

Figure 2B:

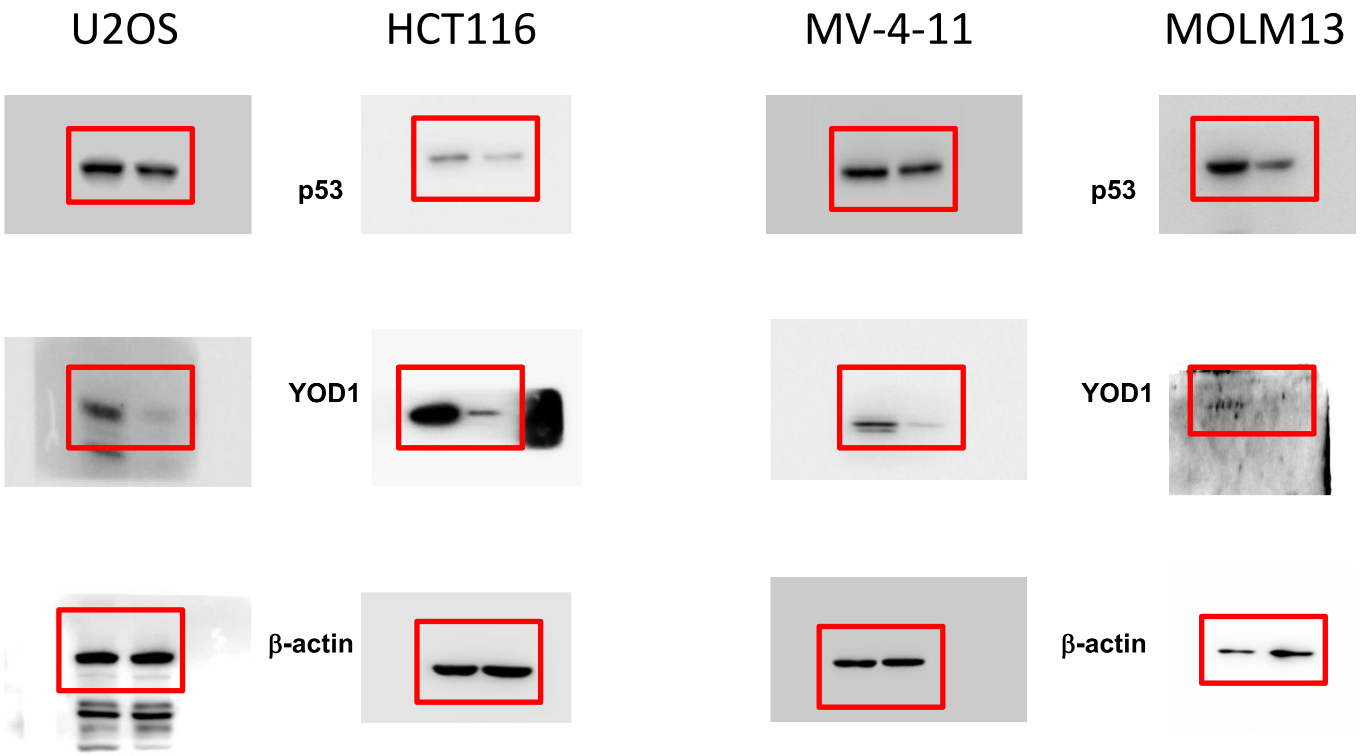

Figure 2C:

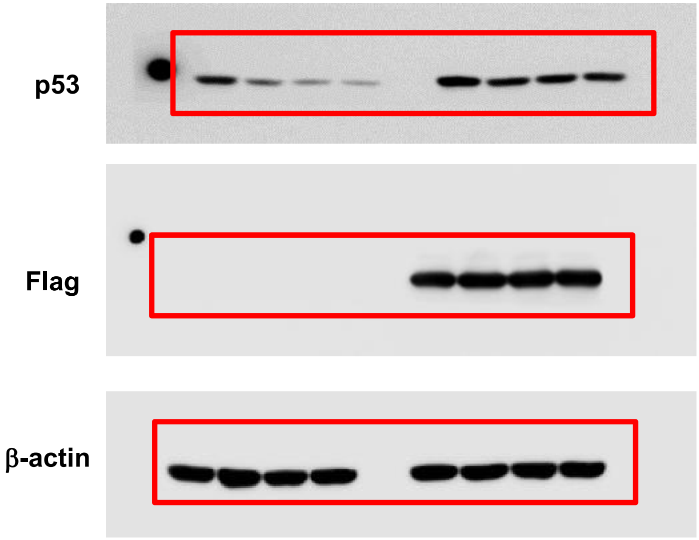

Figure 2D:

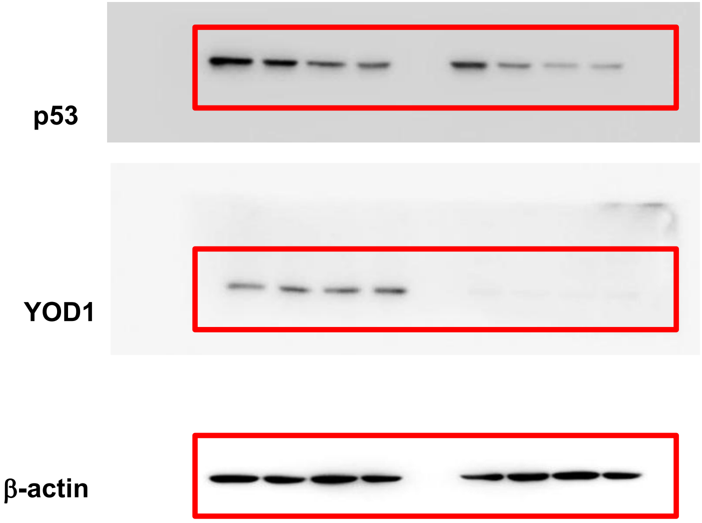

Figure 2E:

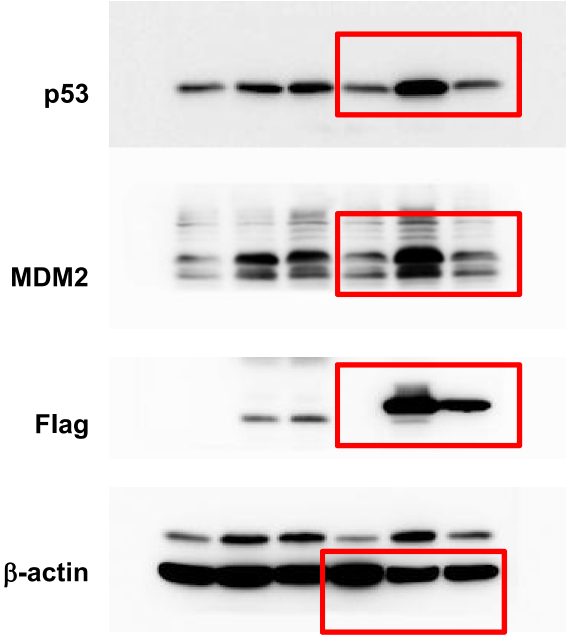

Figure 2F:

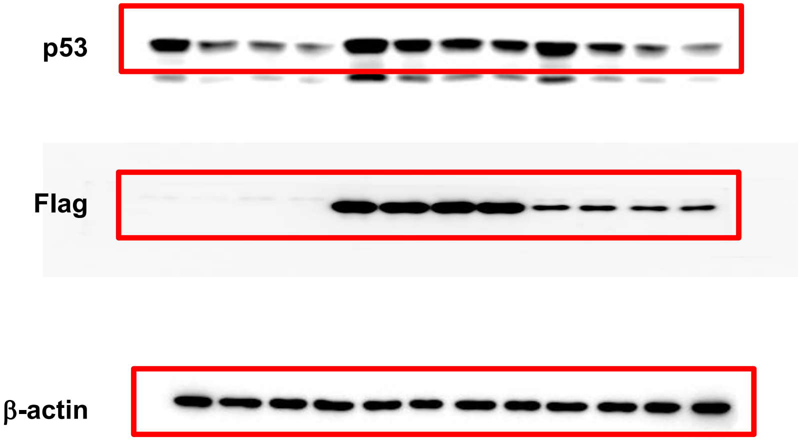

Figure 3A:

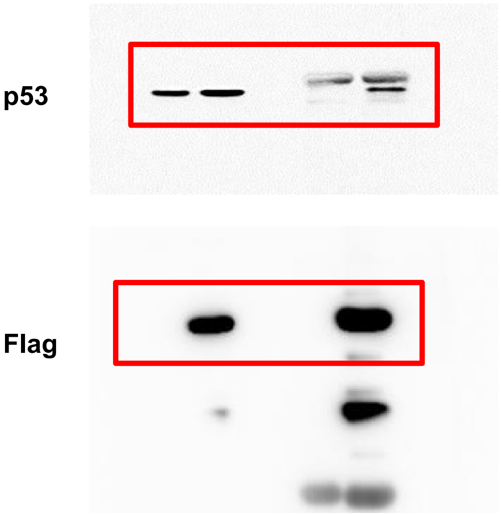

Figure 3B:

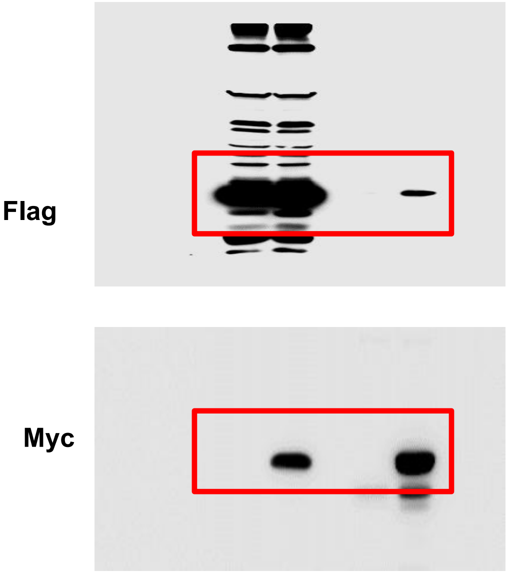

Figure 3C:

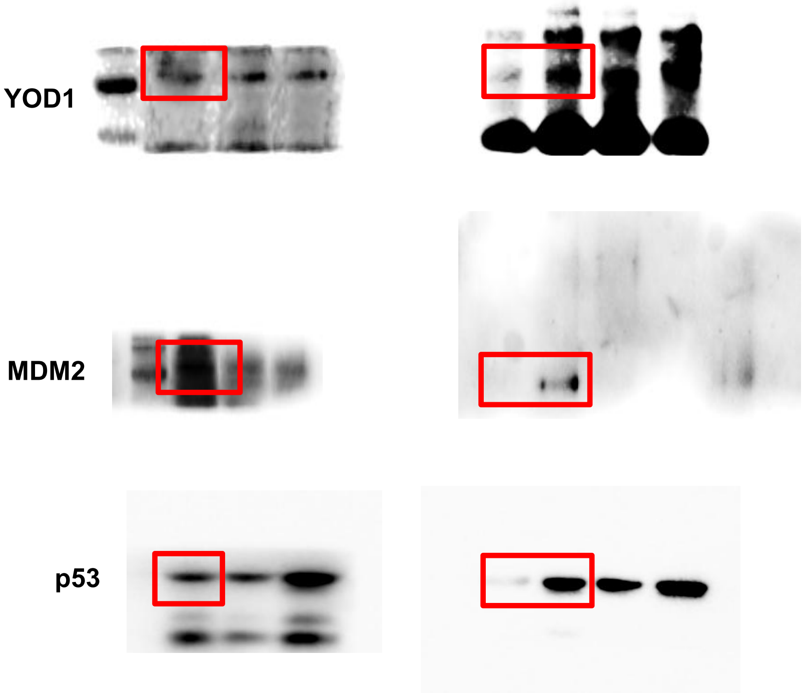

Figure 3D:

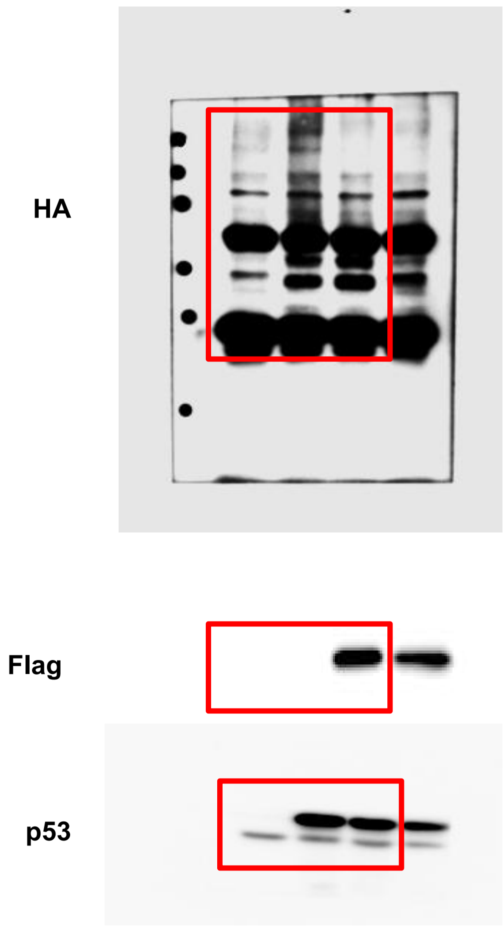

Figure 3E:

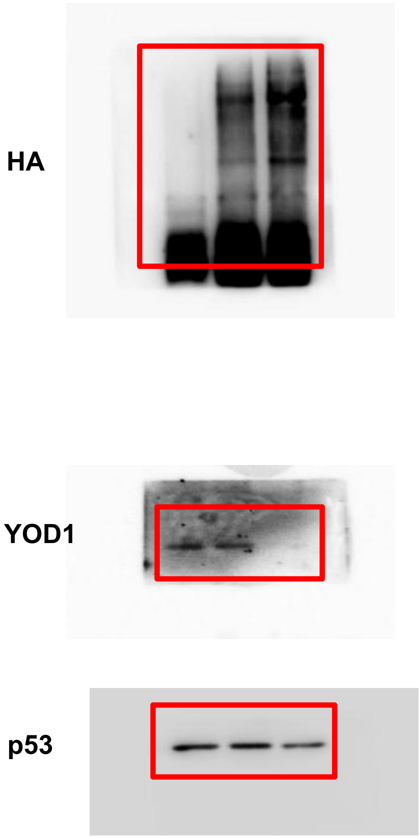

Figure 3F:

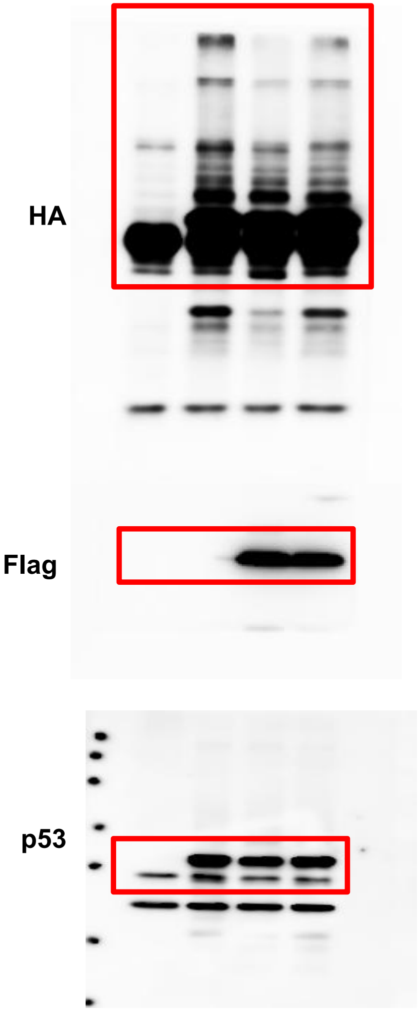

Figure 3G:

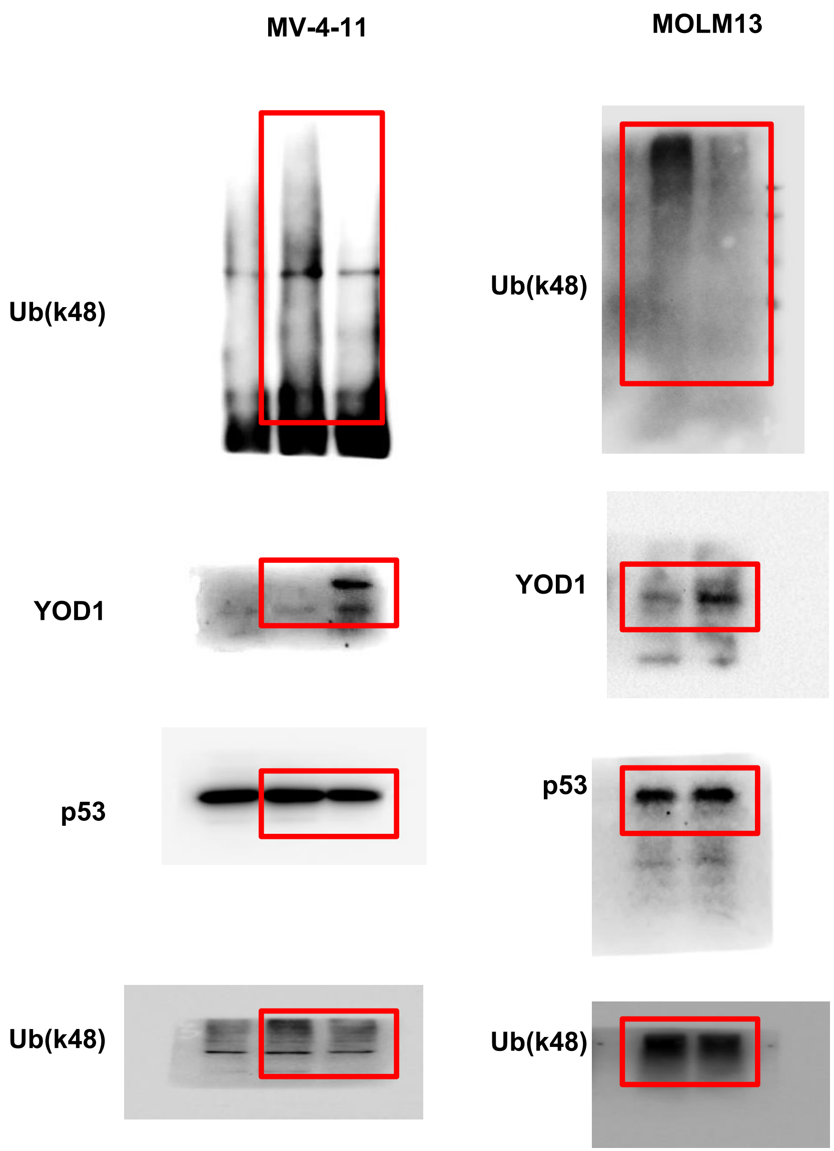

Figure 3H:

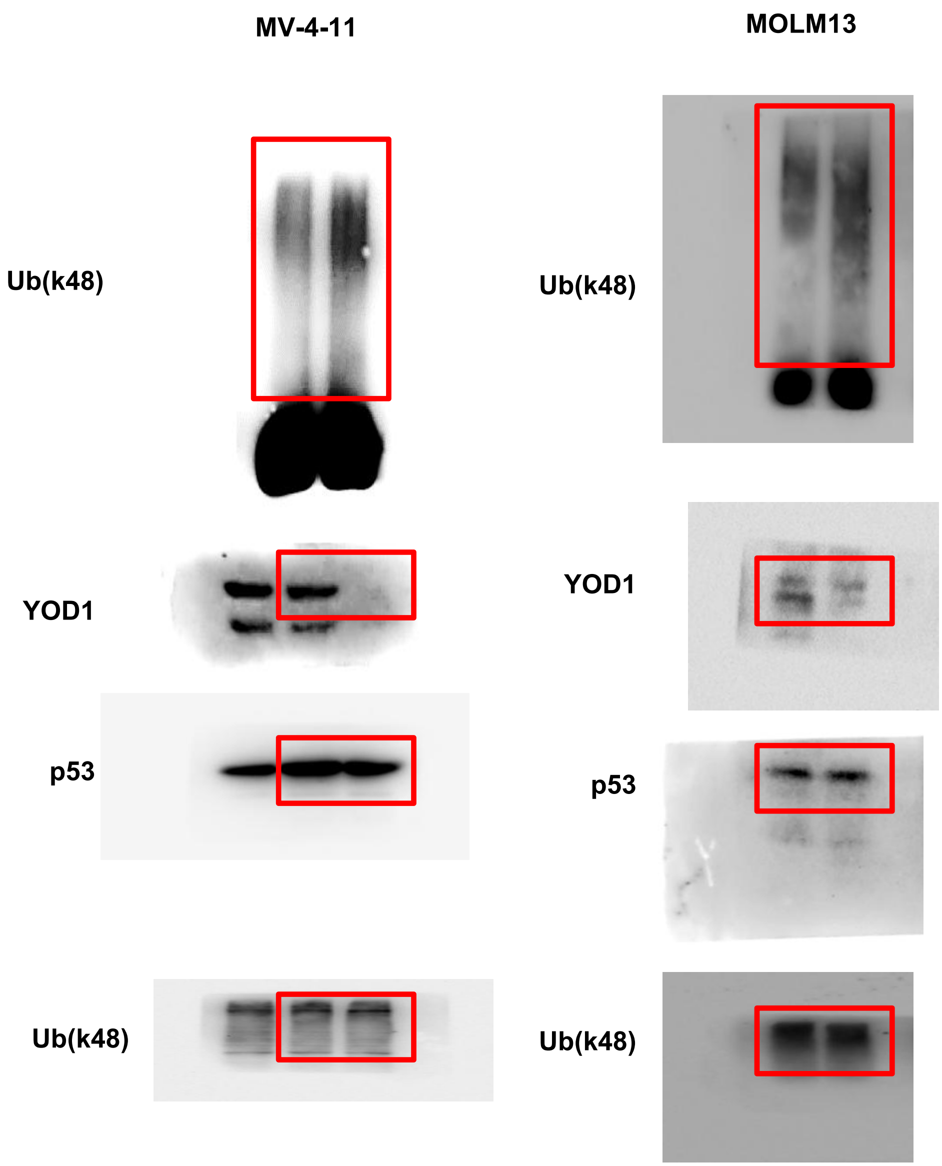

Figure 3I:

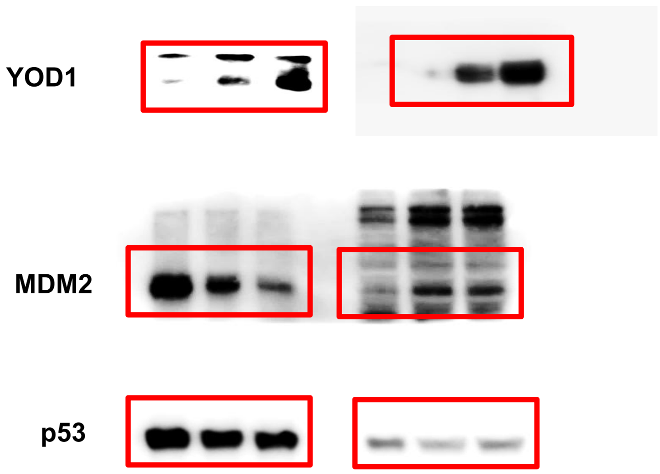

Figure 3J:

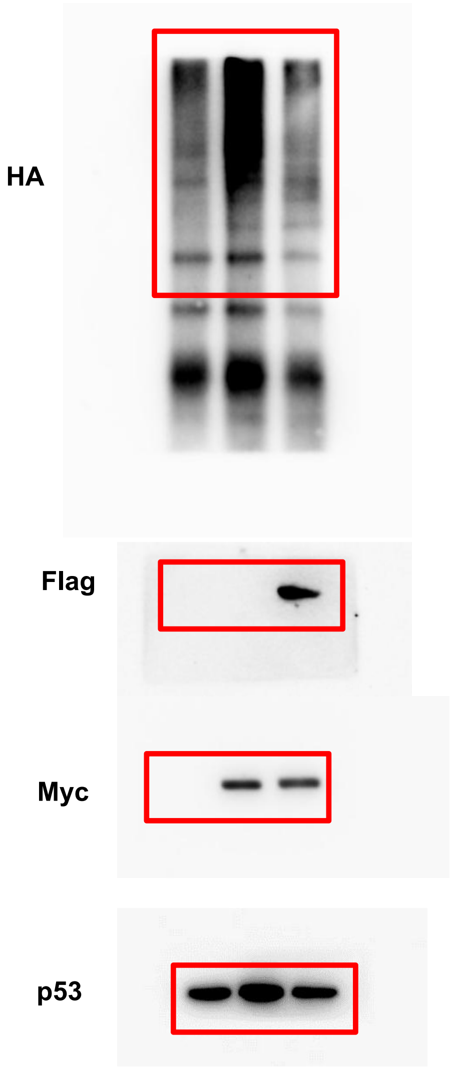

Figure 3M:

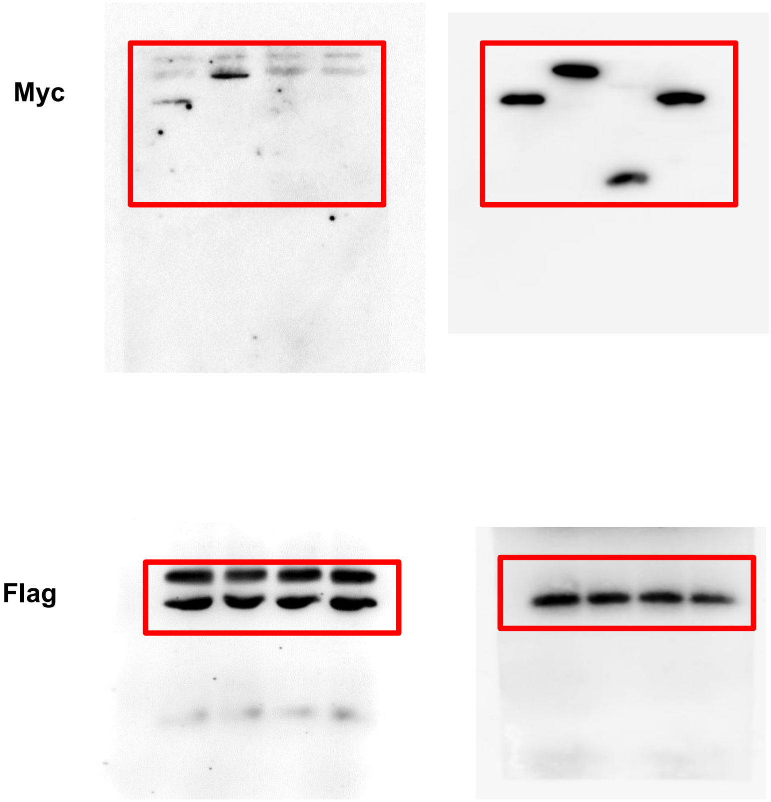

Figure 3O:

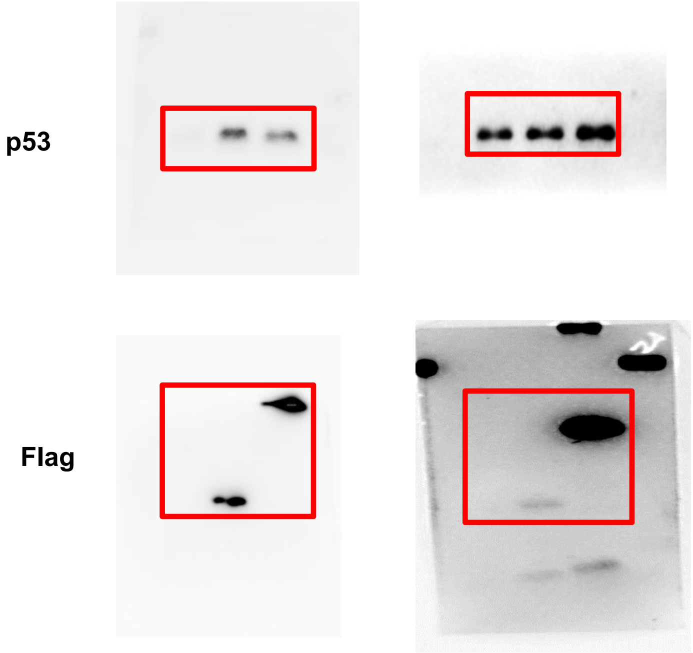

Figure 5A:

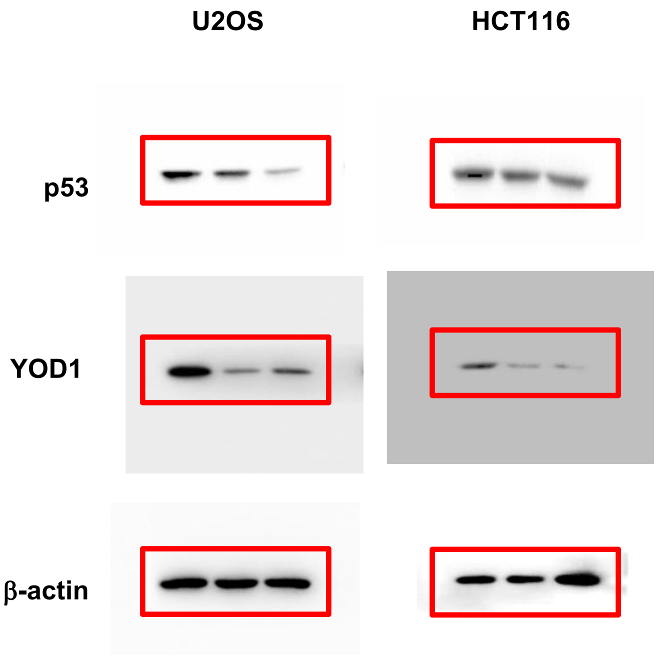

Figure 5B:

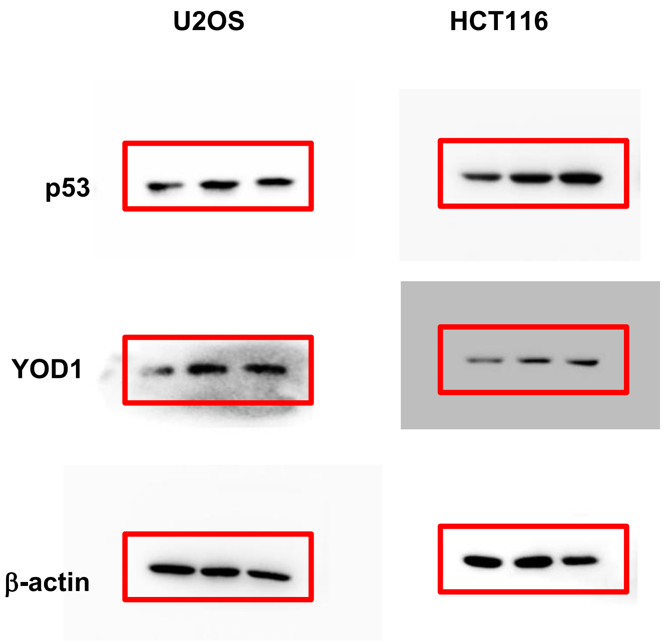

Figure 5C:

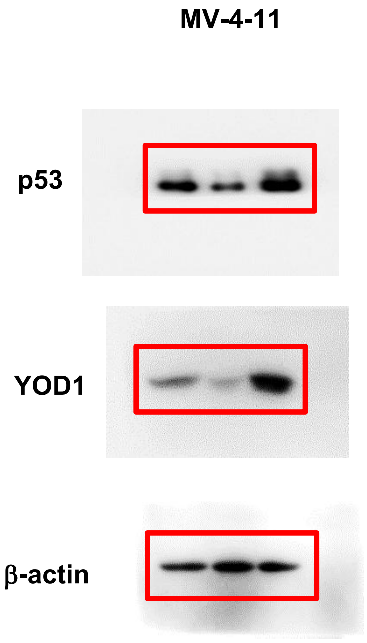

Figure 5D:

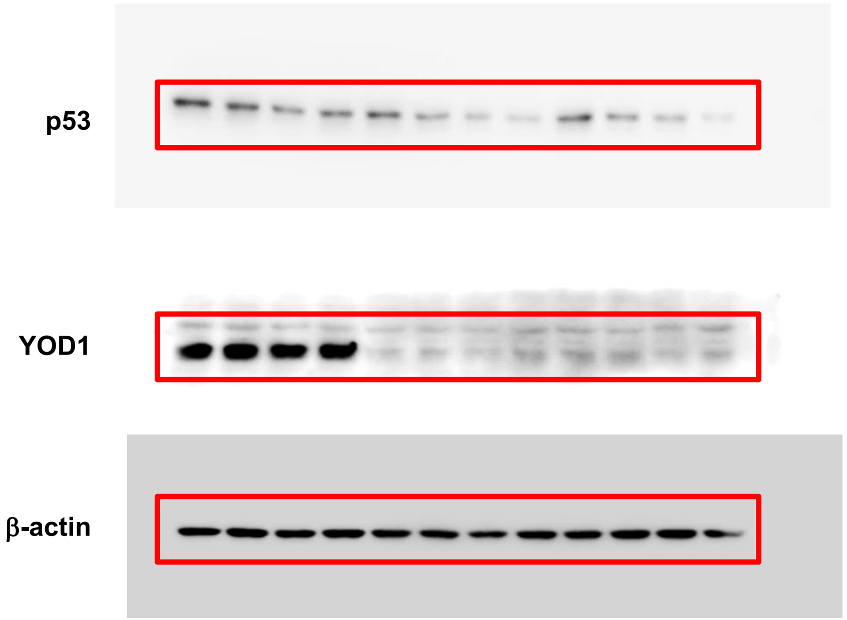

Figure 5E:

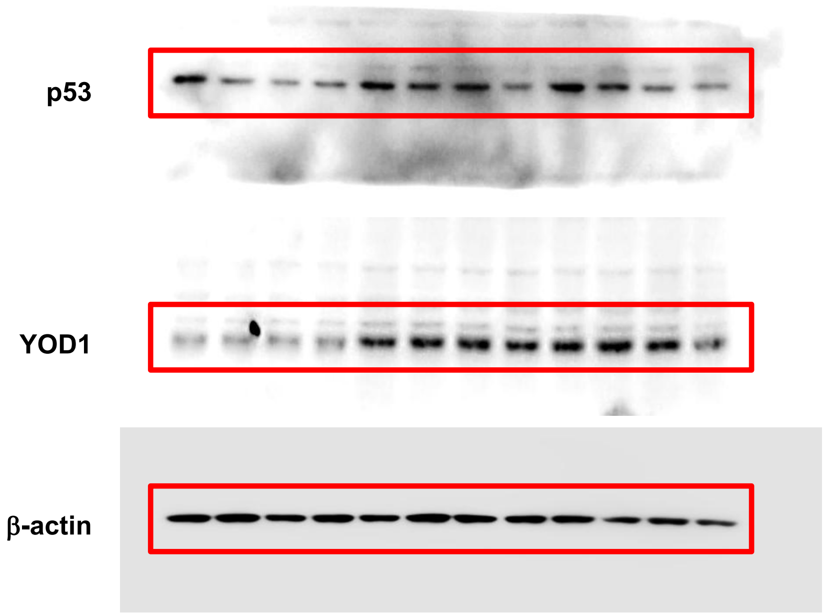

Figure 5F:

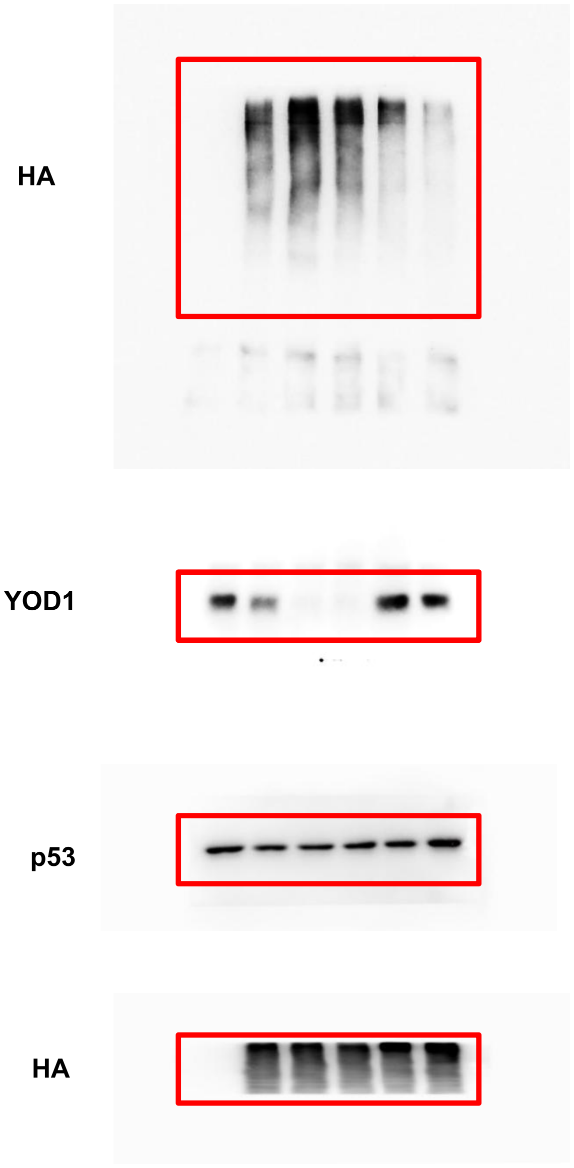

Figure 5G:

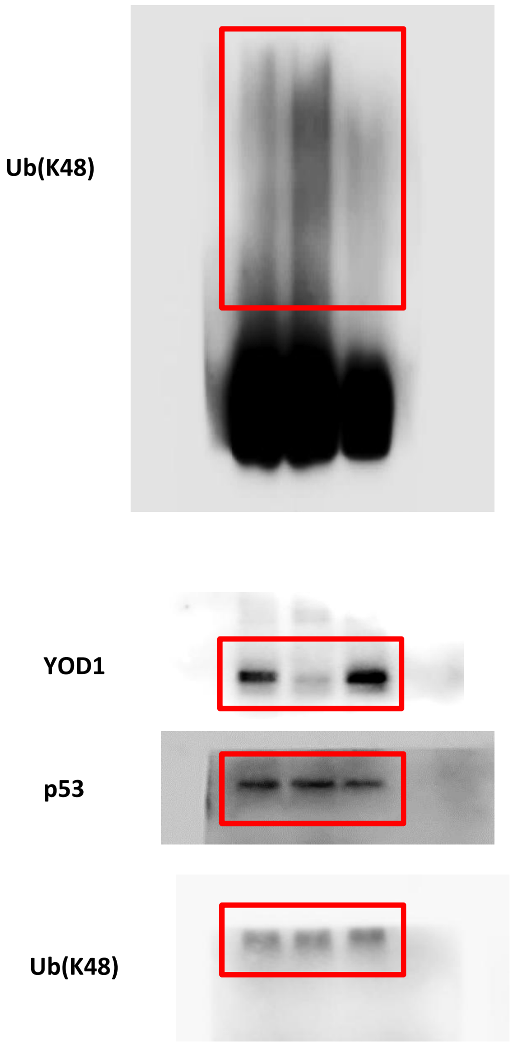

Figure 5H:

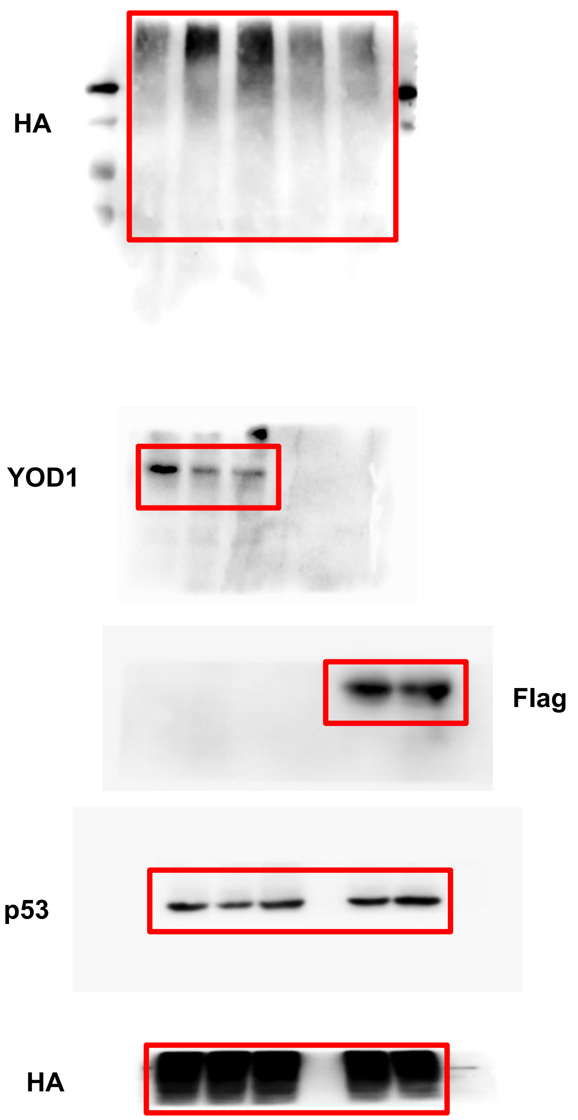

Figure 5I:

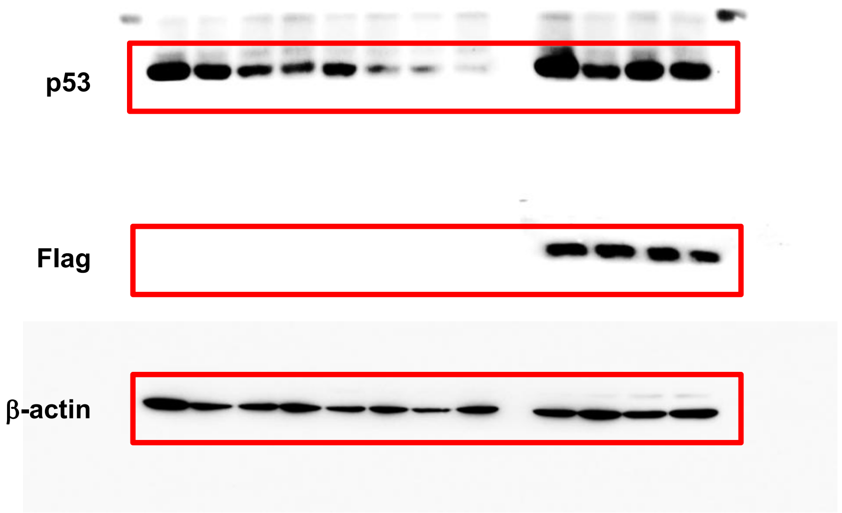

Figure 5J:

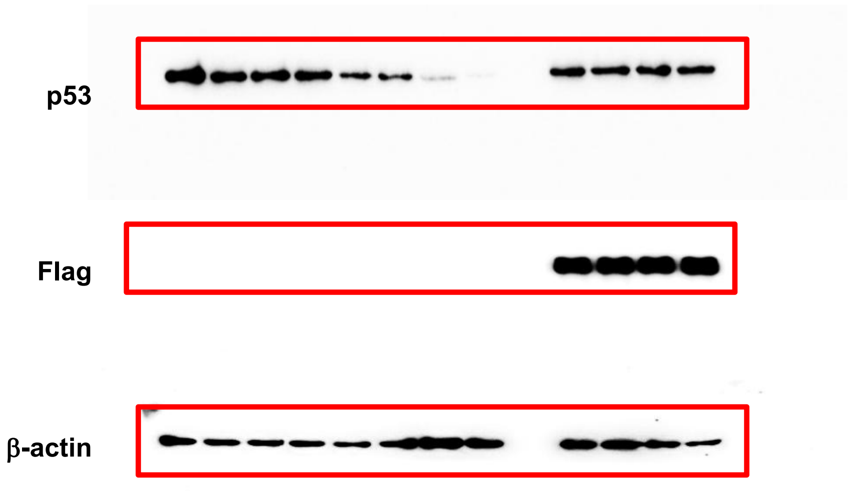

Figure 7A:

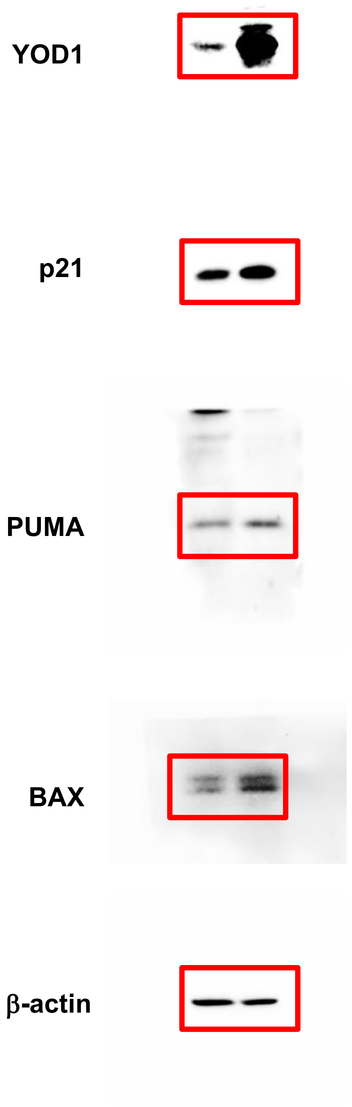

Figure 7B:

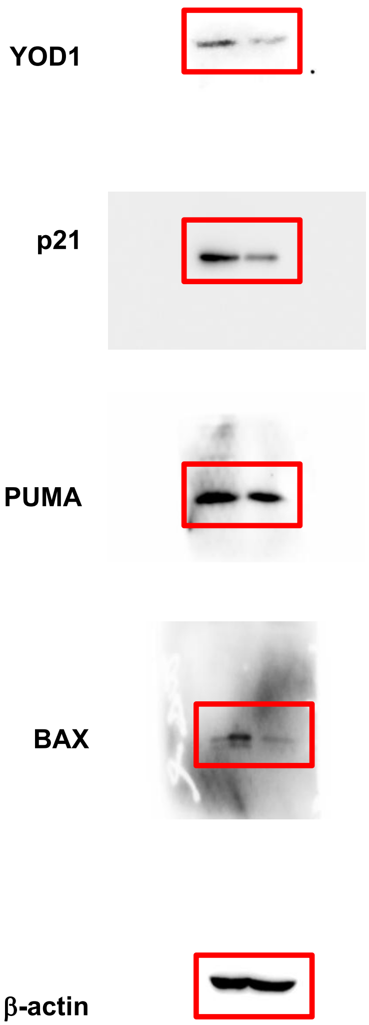

Supplementary Figure 3:

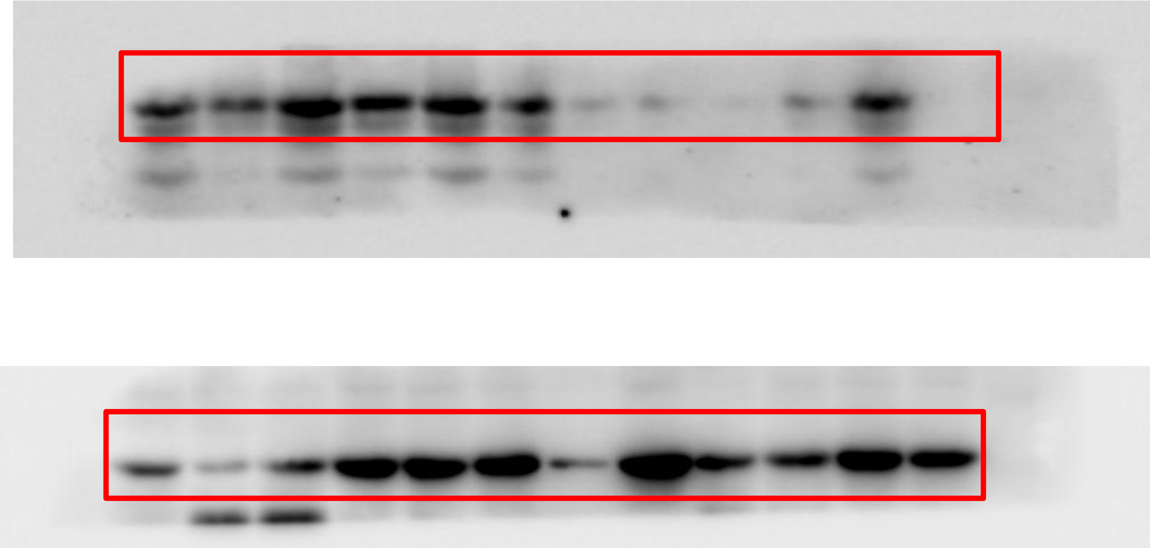

p53

β-actin

Supplementary Figure 5A:

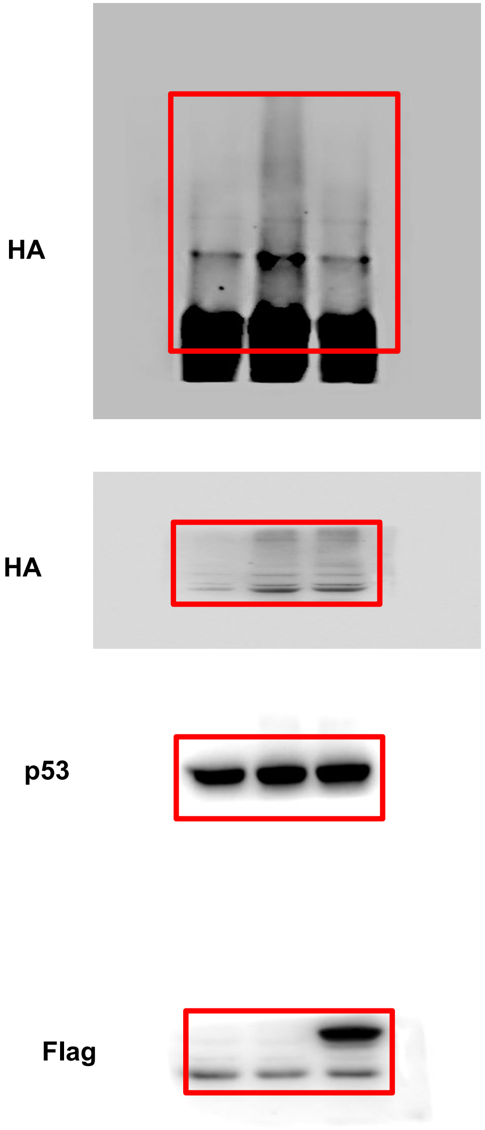

Supplementary Figure 5B:

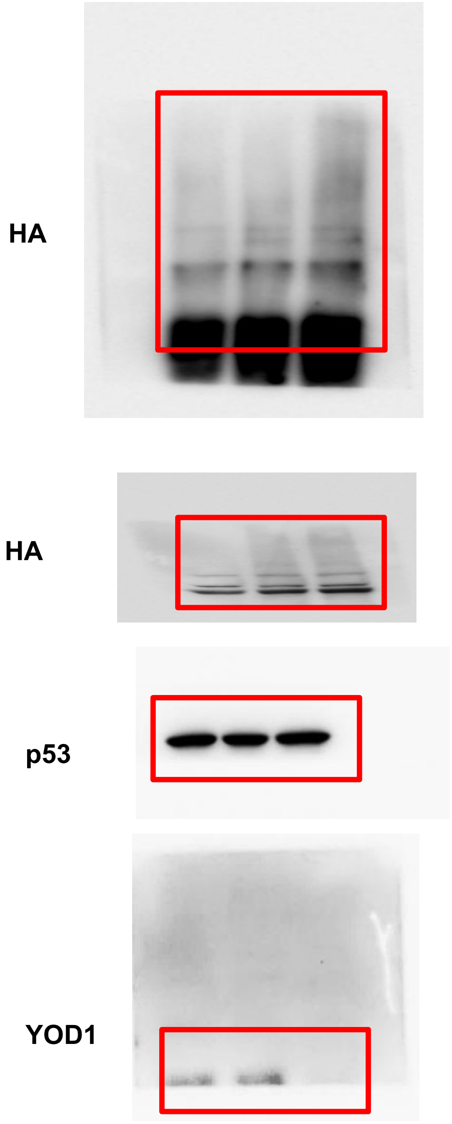

Supplementary Figure 6A :

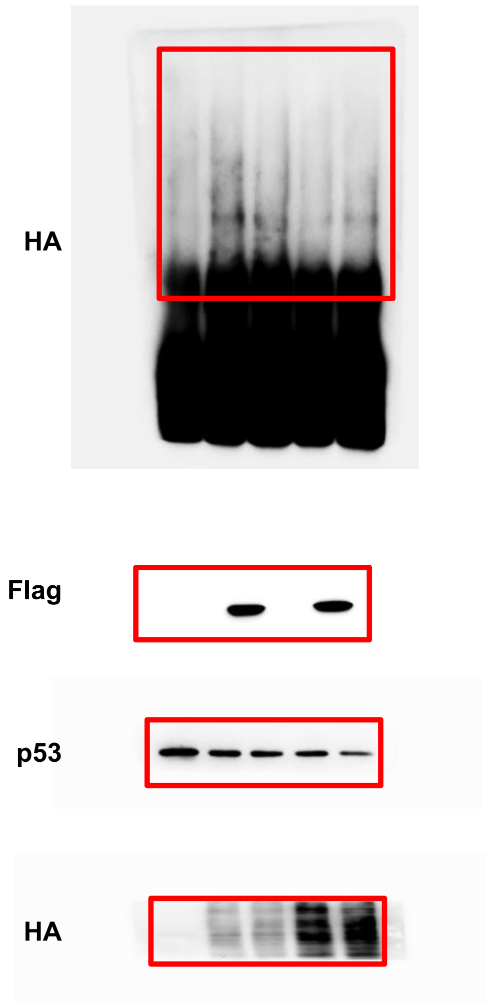

Supplementary Figure 6B:

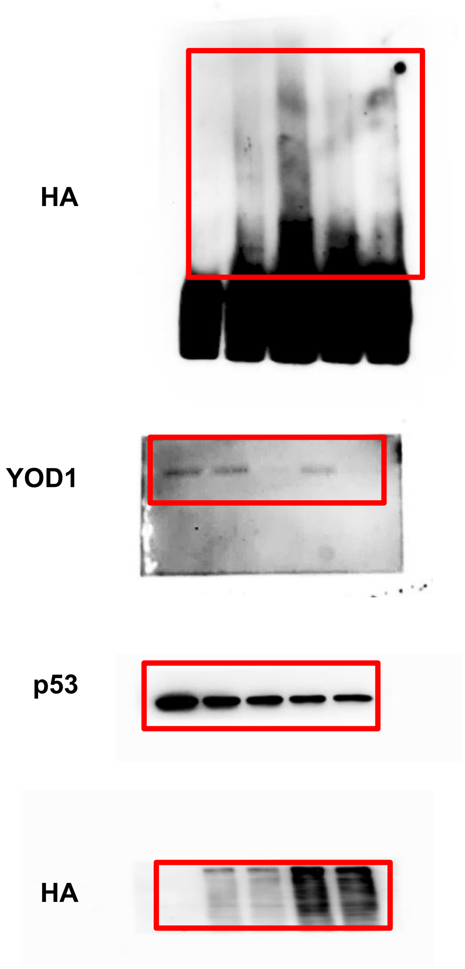

Supplementary Figure 7B:

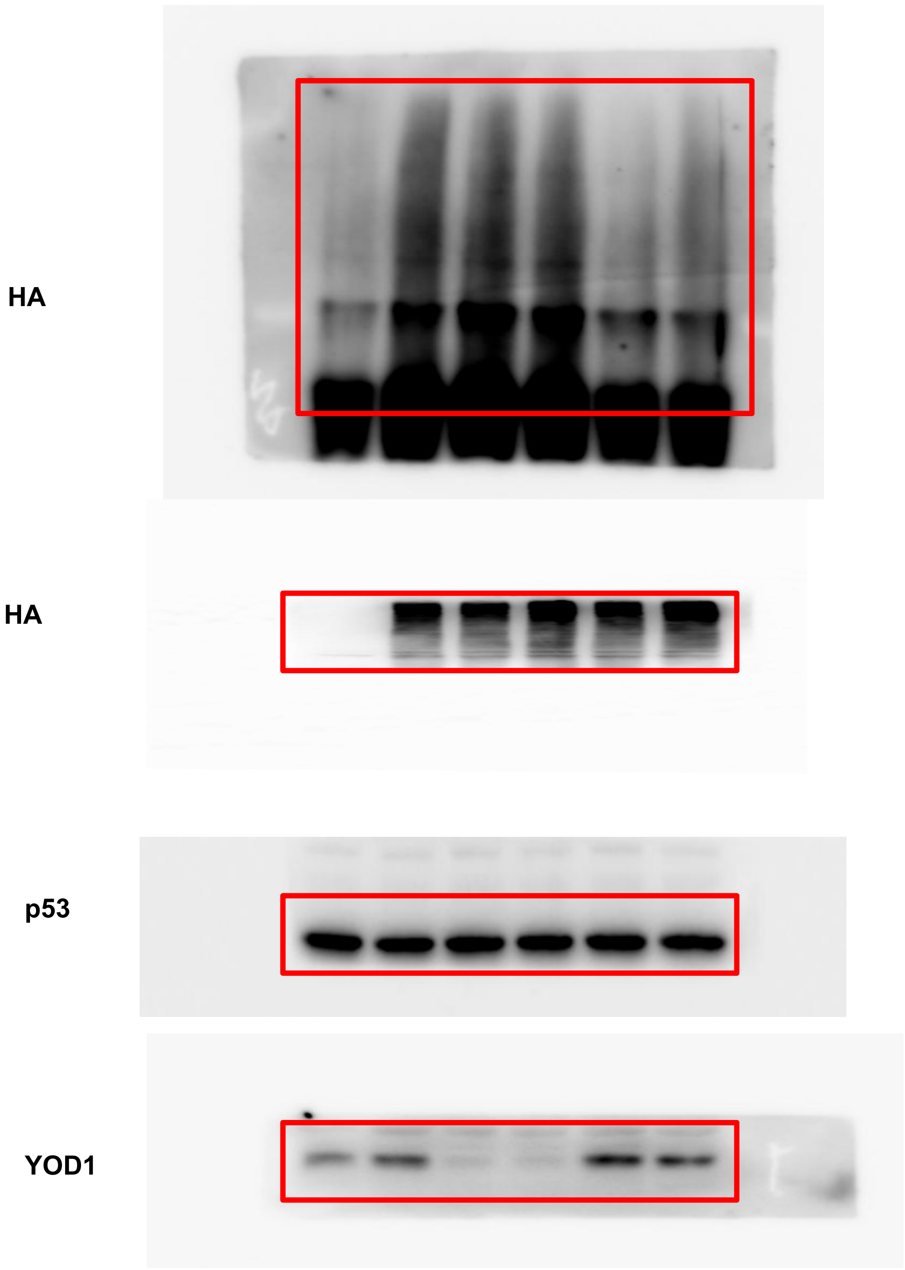

Supplement: Supplementary file 2 — Original Data File [file 41420_2023_1537_MOESM2_ESM.pdf]
