# Supplementary material for: miR-221/222 induce instability of p53 By downregulating deubiquitinase YOD1 in acute myeloid leukemia
Source: Cell Death Discov. 2023 Jul 15;9:249. doi: 10.1038/s41420-023-01537-4 (PMC10349814; doi:10.1038/s41420-023-01537-4)
Supplement: Supplementary file 3 — Certificate of editing [file 41420_2023_1537_MOESM3_ESM.pdf]

editage

Editing Certificate

This document certifies that the manuscript listed below has been edited to ensure language and grammar accuracy and is error free in these aspects. The edit was performed by professional editors at Editage, a division of Cactus Communications. The author's core research ideas were not altered in any way during the editing process. The quality of the edit has been guaranteed, with the assumption that our suggested changes have been accepted and the text has not been further altered without the knowledge of our editors.

MANUSCRIPT TITLE

MiR-221/222 Induce Instability of p53 By Downregulating Deubiquitinase YOD1 in Acute Myeloid Leukemia

AUTHORS

Han Zhong Pei

ISSUED ON

September 05, 2022

JOB CODE

ABGFL_2

THE EDITAGE Promise QUALITY GUARANTEE

Vikas Narang

Vikas Narang
Chief Operating Officer - Editage

editage

Editage, a brand of Cactus Communications, offers professional English language editing and publication support services to authors engaged in over 1300 areas of research. Through its community of experienced editors, which includes doctors, engineers, published scientists, and researchers with peer review experience, Editage has successfully helped authors get published in internationally reputed journals. Authors who work with Editage are guaranteed excellent language quality and timely delivery.

GLOBAL :
+1(833) 979-0061 | request@editage.com

CHINA :
400-120-3020或216-020-9400 |
fabiao@editage.cn

CACTUS

IMPACT SCIENCE impact.science

researcher.life

CACTUS LIFE SCIENCES lifesciences.cactusglobal.com
